# Spring enhancement and summer reduction in carbon uptake during the 2018 drought in northwestern Europe

**DOI:** 10.1098/rstb.2019.0509

**Published:** 2020-09-07

**Authors:** Naomi E. Smith, Linda M. J. Kooijmans, Gerbrand Koren, Erik van Schaik, Auke M. van der Woude, Niko Wanders, Michel Ramonet, Irène Xueref-Remy, Lukas Siebicke, Giovanni Manca, Christian Brümmer, Ian T. Baker, Katherine D. Haynes, Ingrid T. Luijkx, Wouter Peters

**Affiliations:** 1 Meteorology and Air Quality, Wageningen University and Research, Wageningen, The Netherlands; 2 Centre for Isotope Research, University of Groningen, Groningen, The Netherlands; 3 Department of Physical Geography, Faculty of Geosciences, Utrecht University, Utrecht, The Netherlands; 4 Université Aix Marseille, Université Avignon, CNRS, IRD, Institut Méditerranéen de Biodiversité et d'Ecologie marine et continentale (IMBE), Marseille, France; 5 University of Göttingen, Bioclimatology, Büsgenweg 2, 37077 Göttingen, Niedersachsen, Germany; 6 European Commission, Joint Research Centre (JRC), Ispra, Italy; 7 Thünen Institute of Climate-Smart Agriculture, Bundesallee 65, 38116 Braunschweig, Germany; 8 Department of Atmospheric Science, Colorado State University, Fort Collins, CO, USA

**Keywords:** European carbon balance, CO_2_, drought, data assimilation, remote sensing

## Abstract

We analysed gross primary productivity (GPP), total ecosystem respiration (TER) and the resulting net ecosystem exchange (NEE) of carbon dioxide (CO_2_) by the terrestrial biosphere during the summer of 2018 through observed changes across the Integrated Carbon Observation System (ICOS) network, through biosphere and inverse modelling, and through remote sensing. Highly correlated yet independently-derived reductions in productivity from sun-induced fluorescence, vegetative near-infrared reflectance, and GPP simulated by the Simple Biosphere model version 4 (SiB4) suggest a 130–340 TgC GPP reduction in July–August–September (JAS) of 2018. This occurs over an area of 1.6 × 10^6^ km^2^ with anomalously low precipitation in northwestern and central Europe. In this drought-affected area, reduced GPP, TER, NEE and soil moisture at ICOS ecosystem sites are reproduced satisfactorily by the SiB4 model. We found that, in contrast to the preceding 5 years, low soil moisture is the main stress factor across the affected area. SiB4’s NEE reduction by 57 TgC for JAS coincides with anomalously high atmospheric CO_2_ observations in 2018, and this is closely matched by the NEE anomaly derived by CarbonTracker Europe (52 to 83 TgC). Increased NEE during the spring (May–June) of 2018 (SiB4 −52 TgC; CTE −46 to −55 TgC) largely offset this loss, as ecosystems took advantage of favourable growth conditions.

This article is part of the theme issue ‘Impacts of the 2018 severe drought and heatwave in Europe: from site to continental scale’.

## 1. Introduction

The summer of 2018 saw widespread and severe drought in north western Europe. Decreased precipitation in combination with record-high temperatures led to strong reductions in soil moisture availability and decreased atmospheric humidity [1,2]. The combination of high temperatures, low relative humidity, and reductions in soil moisture availability caused plants to close their stomata to conserve water. This led to reductions in evapotranspiration and also photosynthetic activity, thereby reducing carbon uptake from the atmosphere [3,4]. On the other hand, respiration of CO_2_ is typically enhanced with higher temperatures, but during droughts respiration has been shown to decrease owing to a lack of available moisture limiting microbe activity [5,6]. The net impact of these reductions in uptake and release during widespread droughts in Europe, such as in 2003 and 2018, can have a significant impact on the carbon balance of the region.

The terrestrial ecosystems of Europe have been estimated to act as a net sink of carbon. The annual budget comprises a large seasonal cycle of summer uptake and winter loss that is highly sensitive to large-scale weather patterns, and therefore interannual variability is high. Janssens *et al.* [7] estimate an average CO_2_ uptake from the atmosphere between 0.135–0.205 PgC yr^−1^ based on both land-based and atmosphere-based estimates. Luyssaert *et al.* [8] derive a net uptake of 0.20–0.36 PgC yr^−1^ for 2001–2005 following from a combination of atmospheric inverse, inventory and flux measurement-based approaches. Peters *et al.* [9] find a net sink of 0.165 (122–258) PgC yr^−1^ on average in the period 2001–2007 using the atmospheric inverse system CarbonTracker Europe. Monteil *et al.* [10] use a combination of several atmospheric inverse systems and derive a net uptake of 0.21 ± 0.2 PgC yr^−1^ during the 2006–2015 period.

This small net European sink can reduce and even turn into a carbon source during droughts and heatwaves. Ciais *et al.* [5] estimate that the heatwave of 2003 reduced gross primary production (GPP) by roughly 30%, which together with a reduction in total ecosystem respiration (TER) led to a net ecosystem exchange (NEE) of a 0.5 PgC yr^−1^ source of carbon. Peters *et al.* [9] suggest a reduction of the net uptake of 0.147 PgC yr^−1^ over the year 2003, largely neutralizing the carbon sink. Vetter *et al.* [11] estimate a reduction in carbon uptake of 0.02–0.27 PgC yr^−1^. Recently, Buras *et al.* [12] have suggested that the impact of the 2018 drought had an even larger effect on the European carbon balance compared with 2003 based on remote sensing-based observations of the vegetation indices Normalized Difference Vegetation Index (NDVI) and the Enhanced Vegetation Index (EVI).

Severe droughts and heatwaves are expected to occur more frequently in Europe under likely climate change scenarios [13]. It is, therefore, important to understand how the exchange of carbon between the biosphere and the atmosphere is expected to respond to such events. Different ecosystem types respond differently to the effects of droughts and increased temperatures [14] depending on regional geographical characteristics and the ecosystem types present. By using a combination of observational and model-based methods, we aim in this study to cover the impact of the 2018 drought on the European carbon balance from small ecosystem to regional scales. We use inverse modelling and remote sensing to derive European-wide reductions in NEE and GPP respectively, and we use local flux observations and biosphere modelling to distinguish the effects on different ecosystems and understand the processes and drivers of these reductions.

We use observations of eddy-covariance CO_2_ fluxes and atmospheric CO_2_ mole fractions at the stations of the spatially dense Integrated Carbon Observation System (ICOS) [15] together with stations belonging to national projects, and supplemented with remote sensing observations of sun-induced fluorescence (SIF) [16] and vegetative near-infrared reflectance (NIRv) [17], which have both been shown to correlate well with GPP. Previous work has shown the possibility of using SIF to infer the timing and severity of the impact of the 2015/16 El Niño drought on the Amazon region [18], for example, as well as determining the impact and onset of droughts in the USA [19]. Besides these observations, we use the Simple Biosphere model v.4 (SiB4; [20]) to understand the changes in the processes underlying the carbon exchange. This model includes many improvements over its predecessor SiBCASA, which has been shown to often underestimate the soil moisture stress that plants undergo during droughts [21,22]. We will test the ability of SiB4 to simulate the effects of the 2018 drought on the region’s vegetation first as a standalone product and secondly with the approach of van Schaik *et al.* [23], in which the drought response of SiBCASA was improved by coupling it to the more advanced hydrological model PCRaster GLOBal Water Balance (PCR-GLOBWB) [24]. Finally, we use the obtained net biosphere–atmosphere CO_2_ fluxes as a starting point in the data assimilation system CarbonTracker Europe (CTE) [25] and we use atmospheric CO_2_ mole fraction observations to derive an atmospheric view of the reduction in the carbon uptake over Europe during the 2018 drought.

## 2. Methods

**Figure 1.**
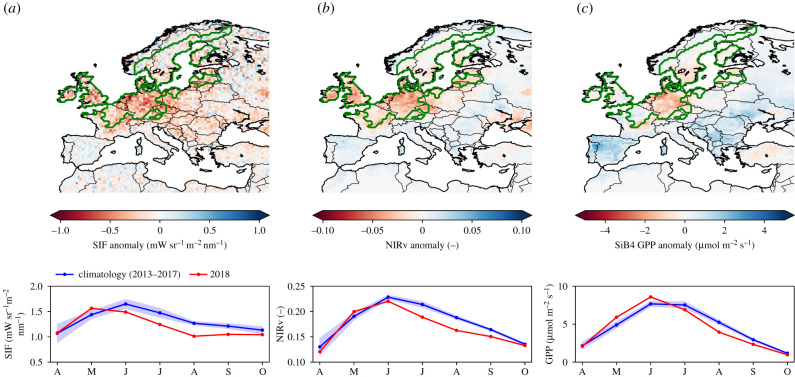
Spatial distribution of mean anomalies during the months July through September 2018 for (*a*) sun-induced fluorescence (SIF) retrieved from GOME-2B, (*b*) near-infrared reflectance of vegetation (NIRv) calculated from MODIS surface reflectance, and (*c*) gross primary production (GPP) simulated by the SiB4 biosphere model. The climatology is based on the same months of 2013–2017. The progression of the climatology and the 2018 anomalies from April to October is shown underneath each map. The green contour indicates the drought-affected area that was based on 2*σ* reduction of MERRA-2 precipitation in the period 2000–2018. (Online version in colour.)

In this section, we describe the observations and methodologies used for this study, throughout which we consider the drought to have had the highest impact during the months July–September of 2018 [1] unless otherwise stated. We study anomalies during this drought period in comparison with the mean for the months July–September for the years 2013–2017, since this is the period for which all data are available. We focus on the northwestern European area as shown in figure 1, encompassed by the contour of a 2*σ* anomaly in precipitation over the period May–September of 2018 relative to the 2000–2017 period. This covers an area of 1.6 × 10^6^ km^2^ over land and corresponds to the ‘extreme drought’ threshold that we are primarily interested in under the system proposed by Quiring [26]. Using the six-monthly Standardized Precipitation Evapotranspiration Index (SPEI) from the SPEI global drought monitor after Bastos *et al.* [27], the same criterion would exclude much of the UK and France, which we know to have been severely affected and which demonstrate strong anomalies in the remote sensing products shown in figure 1. We, therefore, use the precipitation-based mask throughout this paper. We discuss the choice of mask further in electronic supplementary material, §SA.

### (a) Integrated Carbon Observation System ecosystem and atmospheric observations

In this study, we use the homogeneous and standardized dataset from the ICOS network, supplemented by European stations engaged in long-term greenhouse gas monitoring programmes. Eddy-covariance flux measurements are made at the ecosystem sites and CO_2_ mole fractions are measured at the atmospheric sites (see http://www.icos-ri.eu). The current ICOS network consists of 81 ecosystem stations (22 of which have completed the ICOS labelling process) and 36 atmospheric stations (23 fully ICOS-labelled) in 12 countries. In our study, we use the local carbon flux measurements and partitioning thereof into NEE, GPP and TER and compare these with the simulated values from the biosphere model SiB4 (see §2c) [28]. We select ecosystem sites inside and outside the drought region (see electronic supplementary material, table S1 and figure S1). We use monthly NEE (quality flag <0.8) that is filtered using a variable friction velocity threshold and its derived GPP and TER, which are calculated based on night-time partitioning [29]. Atmospheric CO_2_ mole fraction observations are collected at atmospheric stations, including surface (coastal, mountain and a few peri-urban sites), and tall tower sites. [15,30]. In our study, we use these CO_2_ mole fractions to infer carbon fluxes at the surface using the data assimilation system CTE (see §2e). Site information and contacts for both ICOS atmospheric and ecosystem sites can be found in §§A of the electronic supplementary material: tables S1 and S2, respectively.

### (b) Remote sensing of sun-induced fluorescence and near-infrared reflectance

SIF is the small percentage of incident radiation that is re-emitted by chloroplasts during photosynthesis at higher wavelengths, and this signal correlates well with GPP [31]. The retrieved absolute SIF level scales with the intensity of incoming light during the overpass of the satellite. Since we are not interested in this absolute SIF level, which is strongly dependent on the time of overpass, we used photosynthetically active radiation (PAR)-normalized SIF in our analyses. Here, we used SIF from NASA GOME-2B (v.27) which is available from 2013 at a 0.5° × 0.5° spatial resolution [16].

Recently, Badgley *et al.* [17] introduced NIRv, which is calculated as the product of NDVI and total near-infrared reflectance (NIR). This combination addresses the mixed pixel problem that NDVI faces while yielding high-resolution information on the canopy structure and leaf orientation. NIRv has been shown to correlate well with GPP despite being a measure of adjacent processes rather than photosynthesis itself [17]. We use NIRv calculated from MODIS (Moderate Resolution Imaging Spectroradiometer) surface reflectance data adjusted with BRDF (bidirectional reflectance distribution function) [32]. NIRv is available at a high spatial resolution of 0.05° × 0.05°.

We compare the remote sensing observations with the local *in situ* observations of GPP from the ICOS network (§2a), using the SIF and NIRv data subsampled to the location at which the measurements were made. We base this on monthly averages to capture slow changes in productivity, which are more similarly represented by the products than short-term variations [33]. This is the basis for a later upscaling of eddy-covariance inferred GPP [34] in the drought-affected area to a larger domain through NIRv and SIF. The regression of these signals is done per ICOS ecosystem site, and subsequently averaged over forest, grassland and cropland plant-functional types (PFTs) before upscaling. The slopes and uncertainty range we derive and apply in our results are shown in electronic supplementary material, table S3 . Merits and limitations of this method are further discussed in §4.

### (c) Simple Biosphere model v.4

We use the Simple Biosphere model v.4 (SiB4) to simulate biosphere fluxes of carbon during the drought period and preceding years. The SiB model was developed in the ′80s and ′90s with the aim of simulating the land–atmosphere exchange of energy, water and carbon [35,36]). The latest version (SiB4; [37]) integrates previous versions that improved model hydrology (SiB3; [36]), simulated carbon pools (SiBCASA; [38]) and simulated crop phenology (SiBcrop; [39]). The SiB4 model differs from previous versions in the sense that it calculates plant phenology through a process-based model rather than using satellite products to describe the state of the vegetation. The model is run at a spatial resolution of 0.5° × 0.5°.

SiB4 simulates the leaf photosynthesis rate as a minimum of three limiting assimilation rates: that is, (1) limited by the capacity of the RuBisCo enzyme, (2) limited by light, and (3) limited by storage and export in the photosynthesis process [35,40,41]. The SiB4 model simulates different PFTs for specified areal fractions in each grid cell to include land cover heterogeneity [37]. The four most common PFTs used for Europe are: C3-grassland (GRA; non-Tundra), evergreen needleleaf forest (ENF), C3-cropland (CRO) and deciduous broadleaf forest (DBF), covering 14.9, 14.3, 13.8 and 11.9% of the land area, respectively, for the area between −15°E and 40°E longitude and 30°N and 70°N latitude. We will focus our analysis on these four PFTs in Europe.

Meteorological data that are used as forcing for the SiB4 model are taken from the Modern-Era Retrospective Analysis for Research and Applications 2 (MERRA-2) and are available from 1980 onwards [42]. For initialization of the carbon pools, we spin-up the model for the period 1850–1979 until it reaches equilibrium. We incorporate the effect of CO_2_ fertilization to the carbon pools following the observed increase in atmospheric CO_2_ mole fractions [23,43]. The climatological average of MERRA-2 data over the period 1980–2018 is used as meteorological input for the spin-up period from 1850 to 1979. A final simulation is done for 1980–2018 with the actual MERRA-2 driver data of that period. We use a map of fractional PFT coverage for each grid cell based on 0.1° MODIS data [44] and soil characteristics, such as sand and clay fraction, are provided by the International Geosphere–Biosphere Programme (IGBP) Global Soil Data Task Group (2000).

### (d) Soil moisture response in Simple Biosphere model v.4

Previous work has shown that the carbon cycle drought response to soil moisture stress is often not well captured in biosphere models [21,23,45]. While SiB4 already includes several improvements to the model hydrology and has been able to successfully capture drought stress in grassland PFTs over North America [37], we follow two additional strategies to improve the model’s drought response. Firstly, modify the rooting depth in two PFTs to get a stronger expression of soil moisture stress. For ENF and CRO, we find that the default SiB4 rooting depths (4 m) were too deep, which led to constant availability of soil moisture. By assigning shallower rooting depths (1.5 m) to these PFTs based on those of the grass plant functional type (as supported by Yang *et al*. [46]), we see a better drought response in comparison to observations at the ICOS ecosystems sites (see electronic supplementary material, figure S4 for 2018 anomalies of different biosphere fluxes in different SiB4 runs). SiB4 shows quite strong sensitivity to the prescribed lower bounds of the stress factors for ENF PFTs, currently set at 0.7 based on comparisons with North American FLUXNET sites. This high upper bound might not be suitable for European ENF locations (also see electronic supplementary material, figure S4, and the responses described in Lindroth *et al.* [47]). Further work is, therefore, required in order to replicate drought stress across the whole boreal region. In this study, soil moisture and humidity stress factors in needleleaf forests are still minimized at 0.7, as discussed in Haynes *et al.* [48]. Secondly, we have carried out an additional simulation following van Schaik *et al.* [23], in which we coupled SiB4 to the hydrological model PCR-GLOBWB, a two-soil-layer model with full routing scheme and human water use (e.g. irrigation). PCR-GLOBWB provides soil water storage as well as several types of run-off and exchange of water between vertical model components. Full details of the model can be found in Sutanudjaja *et al.* [24]. We have used PCR-GLOBWB to simulate the hydrological system of Europe with a spatial resolution of 0.1° × 0.1° and a daily time step. The meteorological driver data were taken from the ERA5 reanalysis dataset. SiB4 was run with the upper soil layers (0–0.26 m) driven by the top-layer soil moisture output of PCR-GLOBWB (0–0.3 m), instead of its internally calculated soil moisture, following [23]. A comparison of 2018 anomalies of observed and simulated soil water content (SWC) is shown in electronic supplementary material, §SD. This comparison shows that the drought effect in PCR-GLOBWB SWC is limited. Simulated biosphere fluxes therefore do not show an improved drought response relative to the default SiB4 run (electronic supplementary material, figure S4). In §3, we will show results from SiB4 with modified rooting depths and using the default SiB4 soil moisture scheme [48].

### (e) Atmospheric CO_2_ inverse modelling

CTE is a global atmospheric inverse system that estimates the biosphere and oceans’ fluxes of CO_2_, described in full in van der Laan-Luijkx *et al.* [25]. Here, we use atmospheric observations of CO_2_ concentrations from the GLOBALVIEWplus v.5.0 ObsPack product [49] and the European 2018 drought dataset [15,30], made available on the ICOS Carbon Portal, to estimate net carbon fluxes using an ensemble Kalman smoother method. We employ the transport model TM5 [50] on a 3° × 2° grid globally with two-way nested zoom regions of 1° × 1° over North America and Europe. TM5 is driven by ERA-Interim reanalysis meteorology [51].

Two inversions are carried out following this setup: one with prior fluxes for Europe’s terrestrial biosphere taken from SiB4 (§2c) and one with prior fluxes for Europe constructed by taking the climatological mean of the SiB4 fluxes for 2013–2017. For the rest of the globe outside the European area of 30 to 70° N and −15 to 40° E, we used SiBCASA prior fluxes, as in our standard CTE runs [25]. The first of these priors represents our best initial guess of the region’s biosphere fluxes. Using the second, we seek to infer any signal of the drought event purely from the atmospheric observations, with the prior fluxes representing typical conditions for the 5 year period preceding 2018. Prior ocean fluxes are taken from the ocean inversion of Jacobson *et al.* [52]. Scaling factors are optimized for these fluxes on a 1° × 1° resolution with exponentially decaying covariances across each ecoregion, and a length scale of 200 km (see [25]).

Fossil fuel and biomass burning emissions are taken from the EDGAR 4.0 [53] and SiBCASA-GFED4 [54,55] datasets respectively and are not optimized. In 2018, this product exhibits peaks in biomass burning emissions across the south of Sweden, where relatively large wildfires broke out during the dry, hot conditions (see electronic supplementary material, §SF.) We test the sensitivity of the inversion results to the changes we make from our standard CTE setup to one more suitable for investigating extreme drought events. We vary the biosphere prior and observation dataset used.

## 3. Results

### (a) Impact across northwestern Europe

The carbon cycle impact of the 2018 summer drought was recorded in productivity independently across the network of ICOS eddy-covariance observations as well as in remotely sensed SIF and NIRv. Figure 1 shows the anomalies of (*a*) SIF, (*b*) NIRv and (*c*) SiB4 GPP across Europe relative to the 2013–2017 baseline period, with similar patterns of reductions. Strong reductions in SIF and NIRv in northwestern Europe correspond to the area of reduced precipitation (green contour) and high temperatures, contrasting with increases in productivity in eastern Europe and the Iberian peninsula that are also noted in Longdoz *et al.* [56]. Based on 2*σ* reductions in precipitation over land, we estimate the drought-affected area at 1.6 × 10^6^ km^2^, and based on 1*σ* reductions in SIF as well as NIRv, we derive an affected area of 1.9 × 10^6^ km^2^, each with a slightly different spatial distribution. The integrated anomalies for SIF and NIRv are just above 1*σ* in May, become negative starting in June, and drop well below the −1*σ* range in the summer months July–September (JAS).

Within the drought-affected area, 14 out of 16 selected eddy-covariance sites recorded reductions of monthly GPP and/or TER, as derived from the measured NEE in JAS. There was no clear separation of magnitudes across PFTs, nor a strong sign of GPP- or TER-dominated responses. Table 1 shows the changes in the monthly mean carbon balance averaged over PFTs. At most sites (*N*=10 out of 16) and for each of the PFTs, concomitant reductions in TER and (of higher magnitude) GPP lead to overall reduced NEE. Although there is an important role for vegetation stress due to high temperatures, high vapour pressure deficit, and low soil moisture, we find mixed signals of changes in water-use efficiency across sites (not shown) and only evergreen needleleaf forests (ENF) exhibit the clear increase that was demonstrated in Ciais *et al.* [5] and Peters *et al.* [21] during the 2003 drought. Comparison with the SiB4 model results for the same set of sites in table 1 moreover shows that the model captures the magnitude of the anomalies well for deciduous forests, but tends to overestimate the grassland responses and underestimate the drought impact on ENF. At crop sites, SiB4 tends to strongly underestimate the magnitude of the integrated impacts on GPP and TER over the full period, as further discussed in §4.

**Table 1.** Anomalies for the 2018 drought (July–August–September) with respect to the climatological average from 2013 to 2017. GPP, TER and NEE are calculated from monthly mean values and are in the units of gC m^2^ day^−1^. 1*σ* values result from averaging over multiple sites. Note that for the croplands, the crop rotation scheme is not taken into account.

plant functional type	ΔGPP (1σ)	ΔTER (1*σ*)	ΔNEE (1*σ*)
	*eddy-covariance measurements ICOS sites*		
deciduous broadleaf forest (*N* = 4)	−2.46 (3.00)	−1.80 (2.43)	0.63 (2.87)
evergreen needleleaf forest (*N* = 3)	−1.95 (1.93)	−1.31 (1.16)	0.72 (1.02)
C3 grassland (*N* = 4)	−2.16 (3.86)	−1.51 (2.54)	0.40 (1.69)
C3 cropland (*N* = 5)	−1.70 (3.69)	−1.27 (1.12)	0.43 (3.25)
	*SiB4 model sampled at ICOS sites*		
deciduous broadleaf forest (*N* = 4)	−2.49 (1.68)	−1.92 (1.27)	0.57 (0.56)
evergreen needleleaf forest (*N* = 3)	−0.72 (0.40)	0.16 (0.32)	0.88 (0.50)
C3 grassland (*N* = 4)	−3.82 (1.75)	−2.76 (1.07)	1.07 (0.85)
C3 cropland (*N* = 5)	−0.71 (1.47)	−0.01 (0.70)	0.69 (0.89)

### (b) Temporal evolution

**Figure 2.**
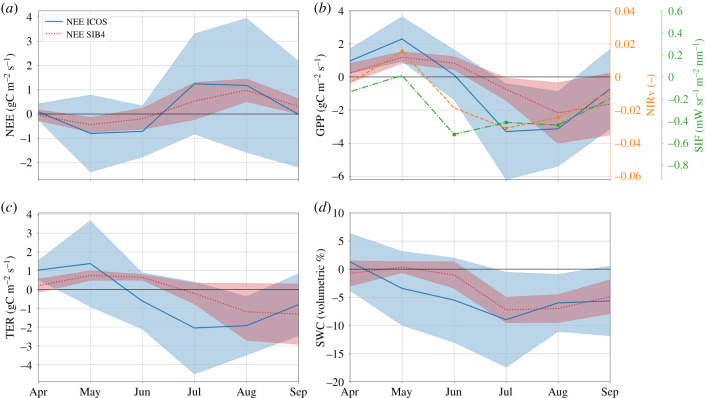
SIF, NIRv and modelled and measured NEE (*a*), GPP (*b*), TER (*c*) and SWC (*d*) anomalies of 2018 against the climatological average (2013–2017) for the seven forest sites (four deciduous broadleaf and three evergreen needleleaf). The average over the different sites is shown together with the 1*σ* spread around the mean. The modelled GPP (SiB4) represents the same PFT as that at the measurement location. SIF and NIRv products are taken from the satellite pixel in which the ICOS measurement site is located and anomalies that exceed 1*σ* are indicated with a cross symbol. Soil moisture is derived from ecosystem site measurements taken in the top 0.05 m of the soil and from the uppermost layers 1–3 of the hydrological component of SiB4. (Online version in colour.)

The summer drought impacts on the carbon cycle were most severe in the July–August–September (JAS) period, and were preceded by favourable growth conditions in spring that increased productivity and net carbon uptake. This is partly visible in the anomalies in figure 1, and further illustrated by the combined deciduous and evergreen forest sites (*N* = 7, grouped together because of their highly similar temporal anomalies) in figure 2 (grassland and crop sites are shown in the electronic supplementary material, §SE). A reduction in summer GPP and TER, as well as NEE (less carbon uptake) is obvious at the forest sites, with the GPP reduction corroborated by locally reduced NIRv and SIF (where the high-resolution NIRv product is in better agreement than the coarser and more noisy SIF signals). SiB4 captures the reductions in ICOS eddy-covariance NEE, GPP, TER and SWC from the forest sites well. The eddy-covariance-data, NIRv and SiB4 all support the positive anomalies in the spring months, likely caused by increases in radiation and temperature while moisture limitations were not yet affecting productivity. Such favourable conditions for spring growth were also seen before a severe summer drought in North America [57], partly offsetting the later loss of carbon from ecosystems. The timing of the transition from this positive anomaly differs between NIRv, SIF, EC-data and SiB4 though, and depends strongly on the progression of soil moisture depletion and the ability of vegetation to access depleting water stores. Figure 2*d* shows this depletion to occur substantially earlier at the ICOS sites than SiB4 simulations suggest. The strongest GPP response consistently occurs once soil moisture goes outside its 1*σ* variability in July.

**Figure 3.**
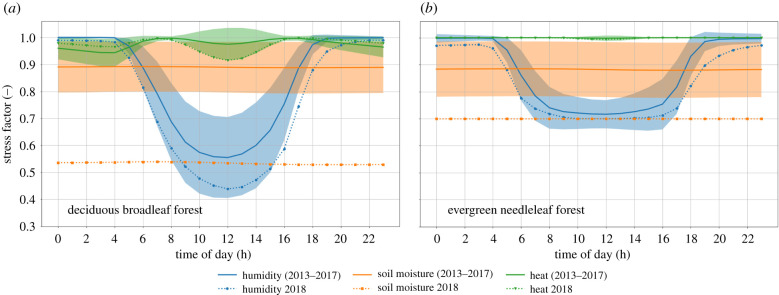
Diurnal cycle of simulated stress factors experienced by (*a*) deciduous broadleaf forest (DBF) and (*b*) evergreen needleleaf forest (ENF) plant functional types for meteorological conditions representative of the German ecosystem site Hainich during the month August. The lowest line indicates that the corresponding stress factor was the one limiting photosynthesis at that point in time, with green showing heat stress, blue humidity stress, and orange soil moisture stress. Solid lines show the climatological mean for 2013–2017 and dotted lines to 2018. The standard deviation is shown in shading for the climatological years. (Online version in colour.)

Figure 3 shows the diurnal cycle of the limiting factors causing stress to vegetation in SiB4 (heat, vapour pressure deficit and soil moisture) for deciduous broadleaf forests (DBF) and evergreen needleleaf forests (ENF) driven using meteorological data for the ecosystem site Hainich, Germany (DE-Hai). For any given time, GPP is limited by the mechanism corresponding to the largest stress (lowest factor) at that moment. For deciduous broadleaf forests, therefore, GPP is limited by soil moisture during the night and vapour pressure deficit in the daytime during climatological years, but was pre-dominantly limited by soil moisture during August 2018. Soil moisture stress in SiB4 scales down the maximum carboxylation rate but also reduces mesophyl conductance, which, in the coupled Ball–Berry–Collatz assimilation conductance scheme, leads to reductions in the ratio of leaf-internal and atmospheric CO_2_ (Ci/Ca), stomatal conductance (gs), and GPP, as well as increased intrinsic water-use efficiency. The same stress factor also scales down heterotrophic respiration (and maintenance respiration), representing reduced microbial activity in dry soils. This makes soil moisture levels, and ensuing stress, a key driver of the biospheric response to severe droughts as studied here. Simulations with an alternative soil moisture distribution derived from the PCR-GLOBWB hydrological model, which performed very well when we applied it to SiBCASA over the Amazon [23], now maintained higher soil moisture and reduced the drought stress, contrasting observations (see electronic supplementary material, figures S5 and S6).

### (c) Integrated anomalies and atmospheric constraints

Regression of the site-specific productivity (*R* = 0.54–0.89 for SIF, and *R* = 0.70–0.97 for monthly NIRv versus eddy-covariance GPP; see electronic supplementary material, §SC) allows PFT-dependent upscaling of NIRv (and SIF) from figure 1 to a total reduction of 203 TgC (340 TgC) over the three-month period with largest impacts (JAS). This can be compared with a third integrated GPP estimate derived from the SiB4 calculations shown in figure 1*c*, which does not use any remotely sensed products to prescribe changes in vegetation phenology. SiB4’s total GPP anomaly integrates to −130 TgC over the same area and time period, as documented in table 2.

**Table 2.** Changes in the European carbon balance (GPP, TER, NEE) in TgC during the period July–September 2018 (JAS), integrated over the northwest European drought-affected area (figure 1), for SIF, NIRv, SiB4 and CTE. Numbers represent deviations from the climatological averages (2013–2017). 1*σ* values for SIF and NIRv result from propagating uncertainty on the fitted slopes (also see electronic supplementary material, table S3). The CTE range is composed of two alternative simulations: one with SiB4 NEE as a prior and the other using the 5-year climatology of SiB4 NEE as a prior. Note that GPP and TER are defined as positive quantities here; thus a negative number represents less gross uptake (GPP) and less gross release (TER), while less net carbon uptake (NEE) is shown by a positive number. The fractional area of the different land-use types is specified relative to the total drought area of 1.6 × 10^6^ km^2^.

	**2018 JAS anomalies in comparison with 2013–2017 mean**					
	GPP			TER	NEE	
aggregation	SIF (1*σ*)	NIRv (1*σ*)	SiB4	SiB4	SiB4	CTE range
forest (28%)	−97 (22)	−56 (19)	−20	−6.0	14	16–24
grassland (22%)	−86 (14)	−55 (12)	−101	−73	28	13–20
crops (27%)	−119 (33)	−69 (18)	−7.9	6.3	14	12–25
other (23%)	−38 (10)	−23 (8)	−1.4	0.2	1.5	11–14
full area	−340 (43)	−203 (30)	−130	−73	57	52–83

**Figure 4.**
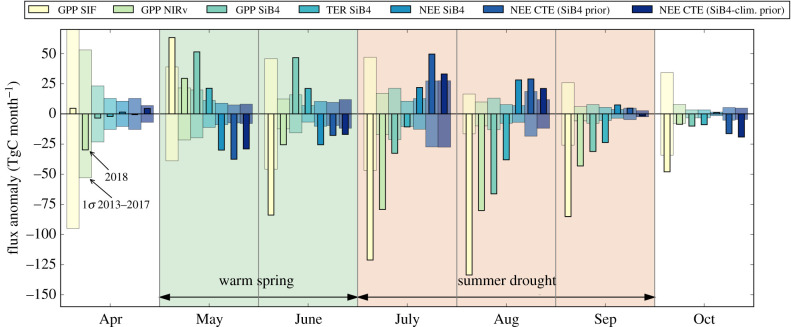
The monthly mean 2018 anomalies compared with the 2013–2017 mean integrated across the 2*σ* precipitation drought mask for: SIF-derived GPP, NIRv-derived GPP, SiB4 GPP, SiB4 respiration (TER), SiB4 NEE, and optimized NEE from CTE from two simulations—one using SiB4 NEE as a prior and the other using the 5-year climatology of SiB4 NEE as a prior. Thinner solid bars show the 2018 anomalies and the wider transparent bars show the 1*σ* standard deviation of the 2013–2017 mean. (Online version in colour.)

The integrated anomaly of TER and NEE calculated with SiB4 amounts to −73 (Ci/Ca) and 57 TgC, respectively, when averaged over the JAS period and the 1.6 × 10^6^ km^2^ drought-affected area indicated in figure 1. This suggests a substantial impact on net carbon exchange that is, as for previous droughts in Europe, a combination of simultaneous GPP and TER (15–25%) reductions. Figure 4 shows the seasonal progression of the SiB4 total anomaly, alongside the independently estimated NIRv and SIF anomaly in GPP. At the integral scale, the contrasting anomalies in spring and summer are even more clearly visible, separating positive and negative impacts on either side of the month of June. The belated response of SiB4 is also visible here though, as simulated GPP in June remains high when NIRv and SIF already suggest reduced productivity.

The integrated NEE anomalies at local to regional scales derived with SiB4 agree well with large-scale constraints derived from atmospheric CO_2_ mole fractions across the ICOS network. Through CTE, we derive a summertime reduction of NEE of 52–83 TgC during JAS, shown alongside the other numbers in table 2. Figure 4 shows the temporal evolution of the anomaly in 2018 starting with the extra net carbon uptake in spring (May–June; −46 to −55 TgC), which quickly turns into the largest cumulative summer carbon loss of the past 5 years by the end of September. The range of the CTE numbers is based on two inversions, one with SiB4 NEE as prior, and the other with the climatological SiB4 NEE as prior. The latter shows that, even without the enhanced spring and reduced summer carbon uptake included in the prior fluxes, the atmospheric inversion derives similar NEE patterns to SiB4. Integrated over the entire year, the annual mean NEE anomaly is 20–49 TgC (i.e. reduced uptake) over the drought-affected region. Over the European TransCom region defined in Gurney *et al.* [58], this annual anomaly becomes −51 to −108 TgC (i.e. additional uptake) owing to the combination of the enhanced spring uptake and an increased uptake over southern and eastern Europe, where more precipitation than usual occurred.

## 4. Discussion

The contrasting carbon cycle anomalies between the spring and summer periods of 2018 strongly resemble the 2012 drought in North America described extensively in Wolf *et al.* [57]. Similarly, during spring 2018 anomalous heat and solar radiation in Europe triggered extra photosynthesis and corresponding evapotranspiration at a time when soil moisture was available to vegetation, leading to extra net carbon uptake. This positive spring anomaly was also detected independently in eddy-covariance data, remote sensing products, biosphere model results, and in the CarbonTracker data assimilation system as constrained by CO_2_ observations from an extensive tower network. As warm conditions persisted, and possibly fed by a positive land–atmosphere feedback discussed in various publications [59–63], soil moisture levels continually decreased to a point where photosynthesis, latent heat release, and to a lesser degree ecosystem respiration, fell well below normal summer values [47,64]. The eventual cancellation of this early spring and late summer anomalous carbon uptake during warmer and dryer years agrees with the analyses of Bastos *et al.* and Kowalska *et al.* [27,65], as well as that of Angert *et al.* [66], who have suggested such a possible trade-off in response to slow climate warming.

Compared with the 2003 drought in Europe, the 2018 impacts on GPP and NEE are large at the site level but integrate to smaller totals, partly because the 2003 event covered a much larger area (3.8 × 10^6^ km^2^, a factor of 2 larger). Also, the 2003 event was preceded by a weaker spring anomaly than in 2018. Sites impacted during both events include Loobos (The Netherlands, PFT: ENF), Tharandt (Germany, PFT: ENF), Hainich (Germany, PFT: DBF) and Sorø (Denmark, PFT: DBF), with needleaf forests showing comparable GPP reductions (approx. 10–15%), and the deciduous forests suggesting much larger reductions (approx. 20–50%) in 2018 than in 2003. The sample size of *N*=2 for both sets precludes strong conclusions on the 2003–2018 difference in drought response of each PFT though, which is more extensively evaluated in Fu *et al.* [67]. In NEE derived from CTE, the 2018 summer reduction of 52–83 TgC in northwestern Europe was compensated for by enhanced spring uptake (−46 to −55 TgC) in the drought-affected area, and also in the wider European region by significantly above-average uptake in southern Europe. On an annual mean integral over all of Europe (TransCom region) this yields a 51–108 TgC net *increase* in net carbon uptake. This contrasts strongly with the 2003 European summer anomaly, which led to a *decreased* European carbon uptake of 147 TgC [9]. This suggests that the strongest effects of the 2018 event were concentrated on a much smaller area. Independent inverse estimates based on the same (sub)set of ICOS atmospheric CO_2_ mole fractions presented in Thompson *et al.* [68] also suggest a positive sign of the summer NEE anomaly (an average of 34% higher than the 10-year mean over northern Europe), and a much lower annual pan-European integrated impact than that of 2003.

We have used the remote sensing product of NIRv, which, unlike SIF, is not a direct measure of photosynthetic activity but bears a close relationship to it [69,70]. NIRv has proven previously [17,33] to correlate more strongly with GPP from eddy-covariance data, and does again in our analyses both during the drought and during the climatological period before. This is likely at least in part due to its higher resolution, which among SIF products can currently only be matched by TROPOMI at a resolution of 7 × 3.5 km daily. TROPOMI SIF was recently used to track photosynthesis across California [71] as well as the dry season of the Brazilian Amazon [72], outperforming NIRv as a GPP-proxy. However, since 2018 was the first year that TROPOMI was operational, we did not investigate the signal from this satellite owing to a lack of preceding data with which to form a climatological period. In previous work [18], we also used a custom SIF product derived from GOME-2A spectra to monitor productivity reductions during a drought. The conversion from SIF to GPP in that work was based on a regression of SIF onto machine-learned [73] and SiBCASA-modelled GPP per PFT, which contrasts with the upscaling based on regression versus eddy-covariance GPP-derivations used here, as well as in Turner *et al.* [71]. In contrast to the latter study, we however make this regression PFT-dependent, as suggested to be needed by various earlier studies [74,75]. We acknowledge that the small number of sites included and relatively short baseline period of 5 years makes this upscaling uncertain though. Furthermore, our initial analysis using regression of only summertime GPP and SIF/NIRv *anomalies* against each resulted in no significant correlation between these anomalies beyond their sign. This once more underwrites the notion that *within* the high-GPP summer regime, the relation between GPP and SIF/NIRv warrants further study.

Our SiB4 model-based analysis suggests that the largest contribution to the total drought anomaly (JAS) in western Europe came from grasslands (NEE: 28 TgC), which had a small measured NEE response per unit area across the ICOS sites in table 1, but which cover the largest area (0.35 × 10^6^ km^2^) of any single PFT in the model while their NEE response is overestimated by SiB4. In agreement with earlier [14] and 2018 drought analyses at eddy-sites [64,76], grassland GPP was the first to show a decline, a response that is captured by the SiB4 model (see electronic supplementary material, figure S8). The forest NEE response shown in figure 2*a* is best reproduced by the model, although it also results from a too late and too small decline in both GPP and TER, and appears to occur at a too small increase of water-use efficiency compared with the (quite noisy) eddy-covariance-derived inherent water-use efficiency. Underestimation of changes in water-use efficiency during droughts is common amongst biosphere models [21], for various reasons discussed in [77]. SiB4-derived anomalies for the crop PFT compared poorly with eddy-covariance observations and to upscaled GPP. This was perhaps to be expected, since the ecosystem sites at which we carried out the comparisons typically rotate the crop type planted annually (e.g. the ICOS site DE-Geb) and each has specific different sowing and growing season lengths, which require specific treatment in crop modelling [78,79]. Like the yield-based analysis of Schrader *et al.* [4], we also found impacts to differ substantially across crop types and sites, especially for NEE. Assessments of crop-specific simulations for Europe in SiB4’s available SiBcrop module [80] are underway, and suggest improvements for maize and wheat are possible with crop-specific parameterizations. Despite remaining shortcomings, the performance increment of SiB4 compared with our previous SiBCASA biosphere model [54] is large and mostly attributable to the better simulation of relative soil moisture levels and the resulting plant stress. Soil moisture is a notoriously difficult parameter to accurately simulate and the agreement between SWC from SiB4, the hydrological model PCR-GLOBWB, and ecosystem site measurements lends credence to the capability of the new simple biosphere model to reproduce extreme soil moisture deficits.

The SiB4 simulated biosphere flux has thereby already made a reasonable prior NEE estimate for the inversions we performed with CTE, but we stress that its outcomes are most strongly driven by the ICOS CO_2_ mole fraction observations. This was confirmed by our simulation using the climatological SiB4 prior, as well as by two alternative inverse simulations with CTE, in which we (a) started from the SiBCASA biosphere model that simulated no 2018 drought anomaly, and (b) additionally removed the extra ICOS CO_2_ observations (*N* = 47 sites, electronic supplementary material, table S2) to fall back on the standard set of sites over Europe. The posterior result of inversion (a) was similar in the timing of the anomaly compared with our CTE simulations shown in figure 4. Also with the SiBCASA prior, we found a sharp transition from negative to positive NEE anomalies from June to July despite starting from only negative summer anomalies in the prior, adding up to a smaller anomaly for JAS (39 TgC instead of 52–83 TgC). Inversion (b) is published as part of the 2019 release of the Global Carbon Project [81], and shows no drought response in JAS and also misses the extra spring uptake. This confirms the important role of the ICOS network to constrain large-scale integrals of NEE from the atmosphere, while at the same time informing on the mechanisms and PFT-specific timing from ecosystem monitoring.

## 5. Conclusion

We estimate that the drought of the summer of 2018 caused a 52–83 TgC drop in the net amount of carbon absorbed by the most strongly affected region in northwestern Europe compared with the climatological mean for July to September. This was partly mitigated by above-average uptake in late spring–early summer and exacerbated by large releases of carbon during January–March, resulting in an annual mean reduction in carbon uptake of 20–49 TgC integrated across the most strongly affected region of 1.6 × 10^6^ km^2^. This strong local response was offset on the European level owing to relatively high amounts of precipitation in southern and eastern Europe, which led to an increase in NEE in these regions. This resulted in a mean annual European net carbon uptake of −51 to −108 TgC, illustrating that there the event was concentrated on a smaller area than that of 2003.

Using the biosphere model SiB4, we have shown that this is primarily due to soil moisture stress, which limited productivity across even typically resilient ecosystems, like evergreen needleleaf forests. Improvements to the hydrological component of this model, as well as corroboration in our findings across remote sensing products, the hydrological model PCR-GLOBWB, and eddy-covariance measurements give us confidence in the NEE anomaly estimates of SiB4. Our subsequent use of it as a high-quality prior estimate in the inverse model CTE, along with constraints from a dense network of observations across the region of interest, confirm the anomaly from the largest perspective.

## Supplementary Material

Supplementary Materials

## Data accessibility

ICOS ecosystem and atmospheric site data are available at the ICOS Carbon Portal (https://www.icos-cp.eu/) [28,30], and the GLOBALVIEWplus v5 ObsPack can be downloaded from the NOAA Earth System Research Laboratory website (https://www.esrl.noaa.gov/gmd/ccgg/obspack/data.php). SIF data are available from the NASA website for the GOME-2B satellite (https://gs614-avdc1-pz.gsfc.nasa.gov/pub/data/satellite/MetOp/GOME_F/v28/MetOp-B/). NIRv, SiB4 and CarbonTracker Europe data created for this study will be available from the ICOS Carbon Portal. The CTDAS code (current revision r1479) is open access under GNU General Public License version 3 and available from the authors. The model PCR-GLOBWB is freely available at https://github.com/UU-Hydro/PCR-GLOBWB_model.

## References

[1] Peters W, Bastos A, Ciais P, Vermeulen A 2020 A historical, geographical and ecological perspective on the 2018 European summer drought. Phil. Trans. R. Soc. B 375, 20190505 (10.1098/rstb.2019.0505) 32892723 PMC7485105

[2] Toreti A *et al.* 2019 The exceptional 2018 European water seesaw calls for action on adaptation. Earth’s Future 7, 652–663. (10.1029/2019EF001170)

[3] Buitink J *et al.* 2020 Anatomy of the 2018 agricultural drought in The Netherlands using *in situ* soil moisture and satellite vegetation indices. Hydrology and Earth System Sciences Discussions 2020, 1–17. (10.5194/hess-2020-358)

[4] Schrader F *et al.* 2020 How exceptionally dry and hot conditions affect carbon fluxes in European croplands. (In preparation).

[5] Ciais P *et al.* 2005 Europe-wide reduction in primary productivity caused by the heat and drought in 2003. Nature 437, 529–533. (10.1038/nature03972) 16177786

[6] Borken W, Savage K, Davidson EA, Trumbore SE 2006 Effects of experimental drought on soil respiration and radiocarbon efflux from a temperate forest soil. Glob. Change Biol. 12, 177–193. (10.1111/j.1365-2486.2005.001058.x)

[7] Janssens IA *et al.* 2003 Europe’s terrestrial biosphere absorbs 7 to 12% of European anthropogenic CO_2_ emissions. Science 300, 1538–1542. (10.1126/science.1083592) 12764201

[8] Luyssaert S *et al.* 2012 The European land and inland water CO_2_, CO, CH_4_ and N_2_O balance between 2001 and 2005. Biogeosciences 9, 3357–3380. (10.5194/bg-9-3357-2012)

[9] Peters W *et al.* 2010 Seven years of recent European net terrestrial carbon dioxide exchange constrained by atmospheric observations. Glob. Change Biol. 16, 1317–1337. (10.1111/j.1365-2486.2009.02078.x)

[10] Monteil G *et al.* Submitted. The regional EUROpean atmospheric transport inversion COMparison, EUROCOM: first results on European wide terrestrial carbon fluxes for the period 2006–2015. Atmos. Chem. Phys. Discuss. (10.5194/acp-2019-1008)

[11] Vetter M *et al.* 2008 Analyzing the causes and spatial pattern of the European 2003 carbon flux anomaly using seven models. Biogeosciences 5, 561–583. (10.5194/bg-5-561-2008)

[12] Buras A, Rammig A, Zang CS 2020 Quantifying impacts of the 2018 drought on European ecosystems in comparison to 2003. Biogeosciences 17, 1655–1672. (10.5194/bg-17-1655-2020)

[13] Samaniego L *et al.* 2018 Anthropogenic warming exacerbates European soil moisture droughts. Nat. Clim. Change 8, 421–426. (10.1038/s41558-018-0138-5)

[14] Teuling AJ *et al.* 2010 Contrasting response of European forest and grassland energy exchange to heatwaves. Nat. Geosci. 3, 722–727. (10.1038/ngeo950)

[15] Ramonet M *et al.* 2020 The fingerprint of the summer 2018 drought in Europe on ground-based atmospheric CO_2_ measurements. Phil. Trans. R. Soc. B 375, 20190513 (10.1098/rstb.2019.0513) 32892733 PMC7485094

[16] Joiner J, Yoshida Y, Guanter L, Middleton EM 2016 New methods for the retrieval of chlorophyll red fluorescence from hyperspectral satellite instruments: simulations and application to GOME-2 and SCIAMACHY. Atmos. Meas. Tech. 9, 3939–3967. (10.5194/amt-9-3939-2016)

[17] Badgley G, Field CB, Berry JA 2017 Canopy near-infrared reflectance and terrestrial photosynthesis. Sci. Adv. 3, 1–6. (10.1126/sciadv.1602244) PMC5362170 28345046

[18] Koren G *et al.* 2018 Widespread reduction in sun-induced fluorescence from the Amazon during the 2015/2016 El Niño. Phil. Trans. R. Soc. B 373, 20170408 (10.1098/rstb.2017.0408) 30297473 PMC6178432

[19] Sun Y, Fu R, Dickinson R, Joiner J, Frankenberg C, Gu L, Xia Y, Fernando N 2015 Drought onset mechanisms revealed by satellite solar-induced chlorophyll fluorescence: insights from two contrasting extreme events. J. Geophys. Res. Biogeosci. 120, 2427–2440. (10.1002/2015JG003150)

[20] Haynes KD, Baker IT, Denning AS, Wolf S, Wohlfahrt G, Kiely G, Minaya RC, Haynes JM 2019 Representing grasslands using dynamic prognostic phenology based on biological growth stages: part 2. Carbon cycling. J. Adv. Model. Earth Syst. 11, 4440–4465. (10.1029/2018MS001541)

[21] Peters W *et al.* 2018 Increased water-use efficiency and reduced CO_2_ uptake by plants during droughts at a continental scale. Nat. Geosci. 11, 744–748. (10.1038/s41561-018-0212-7) 30319710 PMC6179136

[22] van der Molen MK *et al.* 2016 The effect of assimilating satellite-derived soil moisture data in SiBCASA on simulated carbon fluxes in Boreal Eurasia. Hydrol. Earth Syst. Sci. 20, 605–624. (10.5194/hess-20-605-2016)

[23] van Schaik E, Killaars L, Smith NE, Koren G, Van Beek LPH, Peters W, van der Laan-Luijkx IT 2018 Changes in surface hydrology, soil moisture and gross primary production in the Amazon during the 2015/2016 El Niño. Phil. Trans. R. Soc. B 373, 20180084 (10.1098/rstb.2018.0084) 30297478 PMC6178443

[24] Sutanudjaja EH *et al.* 2018 PCR-GLOBWB 2: a 5 arcmin global hydrological and water resources model. Geoscient. Model Dev. 11, 2429–2453. (10.5194/gmd-11-2429-2018)

[25] van der Laan-Luijkx IT *et al.* 2017 The CarbonTracker Data Assimilation Shell (CTDAS) v1.0: implementation and global carbon balance 2001-2015. Geoscientific. Model Dev. 10, 2785–2800. (10.5194/gmd-10-2785-2017)

[26] Quiring SM 2009 Developing objective operational definitions for monitoring drought. J. Appl. Meteorol. Climatol. 48, 1217–1229. (10.1175/2009JAMC2088.1)

[27] Bastos A *et al.* 2020 Direct and seasonal legacy effects of the 2018 heat and drought on European ecosystem productivity. Sci. Adv. 6, eaba2724 (10.1126/sciadv.aba2724) 32577519 PMC7286671

[28] Drought 2018 Team 2019 Drought-2018 atmospheric CO_2_ mole fraction product for 48 stations (96 sample heights) - release 2019-1 (Version 1.0). *ICOS Carbon Portal*. (10.18160/ere9-9d85)

[29] Reichstein M *et al.* 2005 On the separation of net ecosystem exchange into assimilation and ecosystem respiration: review and improved algorithm. Glob. Change Biol. 11, 1424–1439. (10.1111/j.1365-2486.2005.001002.x)

[30] Drought 2018 Team & ICOS Ecosystem Thematic Centre 2019 Drought-2018 ecosystem eddy covariance flux product in FLUXNET-Archive format - release 2019-2 (Version 1.0). *ICOS Carbon Portal* (10.18160/yvr0-4898)

[31] Frankenberg C *et al.* 2011 New global observations of the terrestrial carbon cycle from GOSAT: patterns of plant fluorescence with gross primary productivity. Geophys. Res. Lett. 38, L17706 (10.1029/2011GL048738)

[32] Schaaf C, Wang Z 2015 *MCD43A1 MODIS/Terra+ Aqua BRDF/Albedo Model Parameters Daily L3 Global - 500m V006*. NASA EOSDIS Land Processes DAAC (10.5067/MODIS/MCD43A1.006)

[33] Badgley G, Anderegg LDL, Berry JA, Field CB 2019 Terrestrial gross primary production: using NIR_V_ to scale from site to globe. Glob. Change Biol. 25, 3731–3740. (10.1111/gcb.14729) 31199543

[34] Reichstein M *et al.* 2005 On the separation of net ecosystem exchange into assimilation and ecosystem respiration: review and improved algorithm. Glob. Change Biol. 11, 1424–1439. (10.1111/j.1365-2486.2005.001002.x)

[35] Sellers PJ, Randall DA, Collatz GJ, Berry JA, Field CB, Dazlich DA, Zhang C, Collelo GD, Bounoua L 1996 A revised land surface parameterization (SiB2) for atmospheric GCMs. Part I: model formulation. J. Clim. 9, 676–705. (10.1175/1520-0442(1996)009<0676:ARLSPF>2.0.CO;2)

[36] Baker ET, Prihodko L, Denning AS, Goulden M, Miller S, da Rocha HR 2008 Seasonal drought stress in the Amazon: Reconciling models and observations. J. Geophys. Res.: Biogeosciences 113, G1 (10.1029/2007JG000644)

[37] Haynes KD, Baker IT, Denning AS, Stöckli R, Schaefer K, Lokupitiya EY, Haynes JM 2019 Representing grasslands using dynamic prognostic phenology based on biological growth stages: 1. Implementation in the simple biosphere model (SiB4). J. Adv. Model. Earth Syst. 11, 4423–4439. (10.1029/2018MS001540)

[38] Schaefer K, Collatz GJ, Tans P, Denning AS, Baker I, Berry J, Prihodko L, Suits N, Philpott A 2008 Combined simple biosphere/Carnegie-Ames-Stanford approach terrestrial carbon cycle model. J. Geophys. Res. 113, G03034 (10.1029/2007JG000603)

[39] Lokupitiya E *et al.* 2009 Incorporation of crop phenology in Simple Biosphere Model (SiBcrop) to improve land-atmosphere carbon exchanges from croplands. Biogeosciences 6, 969–986. (10.5194/bg-6-969-2009)

[40] Collatz GJ, Ribas-Carbo M, Berry JA 1992 Coupled photosynthesis-stomatal conductance model for leaves of C_4_ plants. Funct. Plant Biol. 19, 519–538. (10.1071/PP9920519)

[41] Farquhar GD, von Caemmerer S, Berry JA 1980 A biochemical model of photosynthetic CO_2_ assimilation in leaves of C_3_ species. Planta 149, 78–90. (10.1007/BF00386231) 24306196

[42] Gelaro R *et al.* 2017 The Modern-Era Retrospective Analysis for Research and Applications, version 2 (MERRA-2). J. Clim. 30, 5419–5454. (10.1175/JCLI-D-16-0758.1) 32020988 PMC6999672

[43] van der Velde IR, Miller JB, Schaefer K, Masarie KA, Denning S, White JWC, Tans PP, Krol MC, Peters W 2013 Biosphere model simulations of interannual variability in terrestrial _13_C/_12_C exchange. Global Biogeochem. Cycles 27, 637–649. (10.1002/gbc.20048)

[44] Lawrence PJ, Chase TN 2007 Representing a new MODIS consistent land surface in the Community Land Model (CLM 3.0). J. Geophys. Res. 112, G01023 (10.1029/2006JG000168)

[45] Sitch S *et al.* 2008 Evaluation of the terrestrial carbon cycle, future plant geography and climate-carbon cycle feedbacks using five dynamic global vegetation models (DGVMs). Glob. Change Biol. 14, 2015–2039. (10.1111/j.1365-2486.2008.01626.x)

[46] Yang Y, Donohue RJ, McVicar TR 2016 Global estimation of effective plant rooting depth: implications for hydrological modeling. Water Resour. Res. 52, 8260–8276. (10.1002/2016WR019392)

[47] Lindroth A *et al.* 2020 Effects of drought and meteorological forcing on carbon and water fluxes in Nordic forests during the dry summer of 2018. Phil. Trans. R. Soc. B 375, 20190516 (10.1098/rstb.2019.0516) 32892726 PMC7485108

[48] Haynes K, Baker I, Denning S 2020 *Simple biosphere model version 4.2 (SiB4) technical description*. Colorado State University, Libraries. (https://hdl.handle.net/10217/200691)

[49] Cooperative Global Atmospheric Data Integration Project. 2019 *Multi-laboratory compilation of atmosphericcarbon dioxide data for the period 1957–2018;obspack_co2_1_GLOBALVIEWplus_v5.0_2019_08_12, 2019*. See www.esrl.noaa.gov/gmd/ccgg/obspack/data.php?id=obspack_co2_1_GLOBALVIEWplus_v5.0_2019-08-12.

[50] Krol M, Houweling S, Bregman B, van den Broek M, Segers A, van Velthoven P, Peters W, Dentener F, Bergamaschi P 2005 The two-way nested global chemistry-transport zoom model TM5: algorithm and applications. Atmos. Chem. Phys. 5, 417–432. (10.5194/acp-5-417-2005)

[51] Dee DP *et al.* 2011 The ERA-Interim reanalysis: configuration and performance of the data assimilation system. Q. J. R. Meteorol. Soc. 137, 553–597. (10.1002/qj.828)

[52] Jacobson AR, Mikaloff Fletcher SE, Gruber N, Sarmiento JL, Gloor M 2007 A joint atmosphere-ocean inversion for surface fluxes of carbon dioxide: 1. Methods and global-scale fluxes. Glob. Biogeochem. Cycles 21, GB1019 (10.1029/2005GB002556)

[53] European Commission. 2011 *Edgar - Emissions Database for Global Atmospheric Research, release version 4.2*. (EDGAR), European Commission, Joint Research Centre (JRC)/PBL Netherlands Environmental Assessment Agency. See http://edgar.jrc.ec.europa.eu.

[54] van der Velde IR, Miller JB, Schaefer K, van der Werf GR, Krol MC, Peters W 2014 Terrestrial cycling of _13_CO_2_ by photosynthesis, respiration, and biomass burning in SiBCASA. Biogeosciences 11, 6553–6571. (10.5194/bg-11-6553-2014)

[55] Giglio L, Randerson JT, van der Werf GR 2013 Analysis of daily, monthly, and annual burned area using the fourth-generation global fire emissions database (GFED4). J. Geophys. Res. Biogeo. 118, 317–328. (10.1002/jgrg.20042)

[56] Longdoz B *et al.* In preparation. Quantification of 2018 drought for European terrestrial ecosystem plots and impact on parameterisation of CO_2_ fluxes and carbon allocation.

[57] Wolf S *et al.* 2016 Warm spring reduced carbon cycle impact of the 2012 US summer drought. Proc. Natl Acad. Sci. USA 113, 201519620 (10.1073/pnas.1519620113) PMC4889356 27114518

[58] Gurney *et al.* 2002 Towards robust regional estimates of CO_2_ sources and sinks using atmospheric transport models. Nature 415, 626–630. (10.1038/415626a) 11832942

[59] Combe M, Vilà-Guerau de Arellano J, Ouwersloot HG, Peters W 2016 Plant water-stress parameterization determines the strength of land–atmosphere coupling. Agric. For. Meteorol. 217, 61–73. (10.1016/j.agrformet.2015.11.006)

[60] Yin D, Roderick ML, Leech G, Sun F, Huang Y 2014 The contribution of reduction in evaporative cooling to higher surface air temperatures during drought. Geophys. Res. Lett. 41, 7891–7897. (10.1002/2014GL062039)

[61] Miralles DG, Teuling AJ, van Heerwaarden CC, Vilà-Guerau De Arellano J 2014 Mega-heatwave temperatures due to combined soil desiccation and atmospheric heat accumulation. Nat. Geosci. 7, 345–349. (10.1038/ngeo2141)

[62] Fischer EM, Seneviratne SI, Vidale P-L, Lüthi D, Schär C 2007 Soil moisture–atmosphere interactions during the 2003 European summer heat wave. J. Clim. 20, 5081–5099. (10.1175/JCLI4288.1)

[63] GLACE Team *et al.* 2004 Regions of strong coupling between soil moisture and precipitation. Science 305, 1138–1140. (10.1126/science.1100217) 15326351

[64] Graf A *et al.* 2020 Altered energy partitioning across terrestrial ecosystems in the European drought year 2018. Phil. Trans. R. Soc. B 375, 20190524 (10.1098/rstb.2019.0524) 32892732 PMC7485107

[65] Kowalska N, Šigut L, Stojanović M, Fischer M, Kyselova I, Pavelka M 2020 Analysis of floodplain forest sensitivity to drought. Phil. Trans. R. Soc. B 375, 20190518 (10.1098/rstb.2019.0518) 32892727 PMC7485104

[66] Angert A, Biraud S, Bonfils C, Henning CC, Buermann W, Pinzon J, Tucker CJ, Fung I 2005 Drier summers cancel out the CO_2_ uptake enhancement induced by warmer springs. Proc. Natl Acad. Sci. USA 102, 10 823–10 827. (10.1073/pnas.0501647102) PMC1180508 16043702

[67] Fu Z *et al.* 2020 Sensitivity of gross primary productivity to climatic drivers during the summer drought of 2018 in Europe. Phil. Trans. R. Soc. B 375, 20190747 (10.1098/rstb.2019.0747) 32892724 PMC7485099

[68] Thompson RL *et al.* 2020 Changes in net ecosystem exchange over Europe during the 2018 drought based on atmospheric observations. Phil. Trans. R. Soc. B 375, 20190512 (10.1016/j.rse.2018.02.029) 32892731 PMC7485096

[69] Yang P, van der Tol C 2018 Linking canopy scattering of far-red sun-induced chlorophyll fluorescence with reflectance. Remote Sens. Environ. 209, 456–467. (10.1016/j.rse.2018.02.029)

[70] Zeng Y, Badgley G, Dechant B, Ryu Y, Chen M, Berry JA 2019 A practical approach for estimating the escape ratio of near-infrared solar-induced chlorophyll fluorescence. Remote Sens. Environ. 232, 111209 (10.1016/j.rse.2019.05.028)

[71] Turner AJ, Köhler P, Magney TS, Frankenberg C, Fung I, Cohen RC 2020 A double peak in the seasonality of California’s photosynthesis as observed from space. Biogeosciences 17, 405–422. (10.5194/bg-17-405-2020)

[72] Doughty R, Köhler P, Frankenberg C, Magney TS, Xiao X, Qin Y, Wu X, Moore B 2019 TROPOMI reveals dry-season increase of solar-induced chlorophyll fluorescence in the Amazon forest. Proc. Natl Acad. Sci. USA 531, 201908157 (10.1073/pnas.1908157116) PMC6825294 31611384

[73] Beer C *et al.* 2010 Terrestrial gross carbon dioxide uptake: global distribution and covariation with climate. Science 329, 834–838. (10.1126/science.1184984) 20603496

[74] Liu L, Guan L, Liu X 2017 Directly estimating diurnal changes in GPP for C3 and C4 crops using far-red sun-induced chlorophyll fluorescence. Agric. For. Meteorol. 232, 1–9. (10.1016/j.agrformet.2016.06.014)

[75] Magney TS *et al.* 2019 Mechanistic evidence for tracking the seasonality of photosynthesis with solar-induced fluorescence. Proc. Natl Acad. Sci. USA 116, 11 640–11 645. (10.1073/pnas.1900278116) 31138693 PMC6575630

[76] Gharun M *et al.* 2020 Physiological response of Swiss ecosystems to 2018 drought across plant types and elevation. Phil. Trans. R. Soc. B 375, 20190521 (10.1098/rstb.2019.0521) 32892734 PMC7485103

[77] Egea G, Verhoef A, Vidale PL 2011 Towards an improved and more flexible representation of water stress in coupled photosynthesis–stomatal conductance models. Agric. For. Meteorol. 151, 1370–1384. (10.1016/j.agrformet.2011.05.019)

[78] Combe M, de Wit AJW, Vilà-Guerau de Arellano J, van der Molen MK, Magliulo V, Peters W 2017 Grain yield observations constrain cropland CO_2_ fluxes over Europe. J. Geophys. Res. Biogeosci. 122, 3238–3259. (10.1002/2017JG003937)

[79] Lokupitiya E *et al.* 2016 Carbon and energy fluxes in cropland ecosystems: a model-data comparison. Biogeochemistry 129, 53–76. (10.1007/s10533-016-0219-3)

[80] Lokupitiya E *et al.* 2009 Incorporation of crop phenology in simple biosphere model (SiBcrop) to improve land-atmosphere carbon exchanges from croplands. Biogeosciences 6, 969–986. (10.5194/bg-6-969-2009)

[81] Friedlingstein P *et al.* 2019 Global carbon budget 2019. Earth Syst. Sci. Data 11, 1783–1838. (10.5194/essd-11-1783-2019)

