# Supplementary material for: Spring enhancement and summer reduction in carbon uptake during the 2018 drought in northwestern Europe
Source: Philos Trans R Soc Lond B Biol Sci. 2020 Sep 7;375(1810):20190509. doi: 10.1098/rstb.2019.0509 (PMC7485100; doi:10.1098/rstb.2019.0509)
Supplement: Supplementary Materials [file rstb20190509supp1.pdf]

Supplementary Materials for: Spring enhancement and summer reduction in carbon uptake during the 2018 drought in northwestern Europe

Naomi E. Smith[1], Linda M. J. Kooijmans[1], Gerbrand Koren[1], Erik van Schaik[1], Auke M. van der Woude[1,8], Niko Wanders[2], Michel Ramonet[3], Irène Xueref-Remy[3], Lukas Siebicke[4], Giovanni Manca[5], Christian Brümmer[6], Ian T. Baker[7], Katherine D. Haynes[7], Ingrid T. Luijkx[1], and Wouter Peters[1,8]

[1]Meteorology and Air Quality, Wageningen University and Research, Wageningen, The Netherlands

[2]Department of Physical Geography, Faculty of Geosciences, Utrecht University, Utrecht, The Netherlands

[3]Laboratoire des Sciences du Climat et l'Environnement, ICOS, Gif-sur-Yvette, France

[4]University of Göttingen, Bioclimatology, Büsgenweg 2, 37077 Göttingen, Germany

[5]European Commission, Joint Research Centre (JRC), Ispra, Italy.

[6]Thünen Institute of Climate-Smart Agriculture, Bundesallee 65, 38116 Braunschweig, Germany

[7]Department of Atmospheric Science, Colorado State University, Fort Collins, Colorado, USA

[8]Centre for Isotope Research, University of Groningen, Groningen, The Netherlands

Revision 1, May 2020

A Mask of drought-affected area

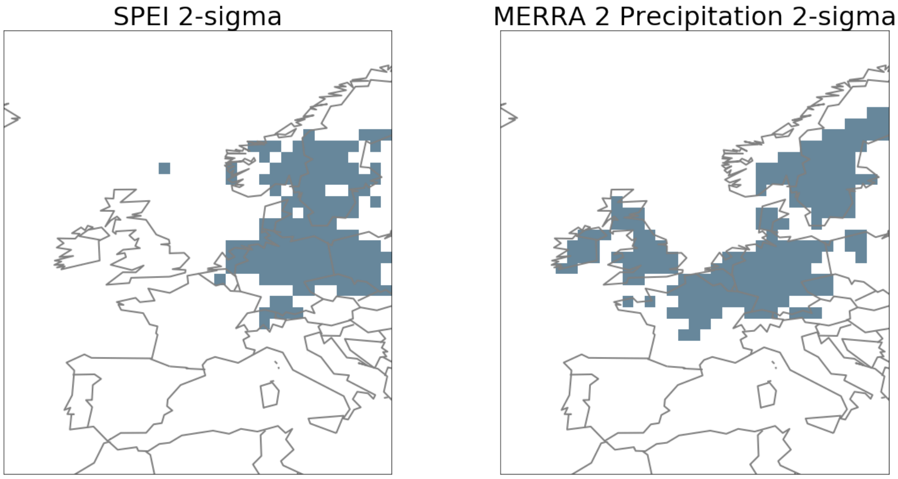

Figure S1: 2-σ mask from the SPEI Global Drought Monitor dataset (1593 thousand km^2) and the smoothed 2-σ MERRA-2 total precipitation mask (1616 thousand km^2) used in the manuscript.

We used a mask based on the total precipitation from the MERRA-2 reanalysis dataset throughout this study. An alternative to this could be a mask based on 6-monthly Standardized Precipitation Evapotranspiration Index (SPEI) from the SPEI Global Drought Monitor dataset, after [1], which would also include information on the temperature and evaporation. Figure S1 shows the masks created using a criterion of 2-σ below the climatology for the two products, chosen to reflect our desire to capture the signal of the area affected by extreme drought [2]. The areas covered are very similar in size, though differ somewhat geographically. The precipitation-based masks extends less far east, but the SPEI-based mask excludes the UK and France entirely, which we see were very strongly effected by the drought in the remote sensing products. The changes to the integrated anomalies using the SPEI-based mask are small and do not change our overall conclusions. for SiB4 GPP, the spring and summer anomalies are very similar (+57.2, -51.4 TgC respectively for SPEI$_{06}$, and +52.3, -50.2 TgC respectively for MERRA-2 precipitation). The percentage changed in raw SIF (NIRv) values between 2018 and the 5-year climatological average are -2.7 % and -4.9 % (-0.2 % and -8.8 %) for spring and summer using the precipitation mask, and -7.3 % and -4.5 % (1.7 % and -7.5 %) for SPEI.

B ICOS sites selected for the drought region

We selected ICOS ecosystem sites within the drought region for evaluation of the SiB4 simulations and for comparison with remote sensing products. The sites were selected based on their ecosystem type, such that they represent either DBF, GRA, ENF and CRO PFTs. To make sure that the observations represent one dominant PFT type in an ecosystem we excluded sites that are classified as mixed forest. Furthermore, the sites had to cover more than 3 years of observations. Table S1 details the sites remaining after the selection procedure, their lat-lon

coordinates, and a literature reference. The locations of these sites are also shown in Figure S2.

Table S1: Selected ICOS ecosystem sites by site code, their coordinates, and a literature reference.

**Site**	**lat** (°N)	**lon** (°E)	**reference**
		DBF	
DE-Hai	51.079200	10.453000	[3]
DE-Hzd	50.964030	13.489820	[4]
DK-Sor	55.485869	11.644644	[5, 6]
FR-Hes	48.674100	7.064650	[7]
		GRA	
DE-Gri	50.950000	13.512600	[4]
DE-Rur	50.621917	6.304139	[8]
CH-Fru	47.115800	8.537800	[9]
CH-Cha	47.210200	8.410400	[10]
		ENF	
NL-Loo	52.166581	5.743556	[11]
SE-Htm	56.097630	13.418970	[12]
DE-Tha	50.962400	13.565200	[4]
		CRO	
BE-Lon	50.551600	4.746100	[13]
DE-Geb	51.099730	10.914630	[14, 15]
DE-Kli	50.893100	13.522400	[4]
DE-Rus	50.865907	6.447145	
SE-Lnn	58.340630	13.101768	

From the atmospheric ICOS measurement sites, we assimilate data from each location, the highest intake-level of a tower if available. Table S2 summarises the sites available, their locations, and points of contact.

Table S2: Atmospheric sites for which data was made available for the drought period via the ICOS atmospheric observations network.

**Site code**	**Site name**	**Latitude** (°N)	**Longitude** (°E)	**Site Contacts**
BIK	Bialystok	53.22	23.03	Christoph Gerbig<br>Jost Lavric
BIR	Birkenes Observatory	58.39	8.25	Cathrine Lund Myhre<br>Ove Hermansen
BIS	Biscarrosse	44.38	-1.23	Marc Delmotte<br>Michel Ramonet
BRM	Beromunster	47.1896	8.1755	Markus Leuenberger
BSD	Bilsdale	54.359	-1.155	Simon ODoherty<br>Dan Say
CBW	Cabauw	51.971	4.9275	Arnoud Frumau<br>Arjan Hensen<br>Alex Vermeulen
CMN	Monte Cimone	44.17	10.68	Luigi Caracciolo di Torchiarolo<br>Paolo Cristofanelli
CRP	Carnsore Point	52.18	-6.375	Damien Martin<br>Colin O'Dowd
DEC	Delta de l'Ebre	40.7439	0.7867	Josep-Anton Morgui<br>Roger Curcoll
EEC	El Estrecho	36.0586	-5.664	Josep-Anton Morgui<br>Roger Curcoll
ERS	Ersa	42.9692	9.3801	Marc Delmotte<br>Carole Philippon<br>Michel Ramonet
FKL	Finokalia	35.3378	25.6694	Marc Delmotte<br>Nikos Mihalopoulos<br>Michel Ramonet

Table S2: Atmospheric sites for which data was made available for the drought period via the ICOS atmospheric observations network.

Site code	Site name	Latitude (°N)	Longitude (°E)	Site Contacts
GAT	Gartow	53.0657	11.4429	Dagmar Kubistin<br>Matthias Lindauer<br>Christian Plass-Duelmer<br>Dietmar Weyrauch
GIC	Sierra de Gredos	40.3457	-5.1755	Joesp-Anton Morgui<br>Roger Curcoll
HEI	Heidelberg	49.417	8.674	Ingeborg Levin<br>Samuel Hammer
HPB	Hohenpeissenberg	47.8011	11.0246	Dagmar Kubistin<br>Matthias Lindauer<br>Christian Plass-Duelmer<br>Dietmar Weyrauch
HTM	Hyltemossa	56.0976	13.4189	Michal Heliasz<br>Jutta Holst
HUN	Hegyhatsal	46.95	16.65	Laszlo Haszpra
IPR	Ispra	45.8147	8.636	Giovanni Manca<br>PEter Bergamaschi
IZO	Izana, Tenerife,<br>Canary Islands	28.309	-16.499	Emilio Cuevas-Agulló
JFJ	Jungfraujoch	46.55	7.987	Martin Steinbacher
KAS	Kasprowy Wierch,<br>High Tatra	49.2325	19.9818	Jarosław Necki<br>Lukasz Chmura
KRE	Křešín u Pacova	49.572	15.080	Kateřina Komínková<br>Michal V. Marek<br>Gabriela Vítková
LHW	Laegern-Hochwacht	47.4822	8.3973	Dominik Brunner<br>Stephan Henne

Table S2: Atmospheric sites for which data was made available for the drought period via the ICOS atmospheric observations network.

**Site code**	**Site name**	**Latitude** (°N)	**Longitude** (°E)	**Site Contacts**
LIN	Lindenberg	52.1663	14.1226	Dagmar Kubistin<br>Matthias Lindauer<br>Christian Plass-Duelmer<br>Dietmar Weyrauch
LMP	Lampedusa	35.53	12.52	Alcide Giorgio di Sarra<br>Salvatore Piacentino<br>Damiano Sferlazzo
LMU	La Muela	41.5941	-1.1003	Josep-Anton Morgui<br>Roger Curcoll
LUT	Lutjewad	53.4036	6.3528	Huilin Chen<br>Bert Scheeren
MHD	Mace Head	53.3261	-9.9036	Marc Delmotte<br>Damien Martin<br>Michel Ramonet<br>Gerard Spain
MLH	Malin Head	55.355	-7.333	Damien Martin<br>Colin O'Dowd
NOR	Norunda	60.0864	17.4794	Jutta Holst<br>Irene Lehner<br>Meelis Molder
OHP	Observatoire de Haute Provence	43.931	5.712	Pierre-Eric Blanc<br>Marc Delmotte<br>Michel Ramonet<br>Irene Xueref-Remy
OPE	Observatoire pérenne de l'environnement	48.5619	5.5036	Sebastien Conil<br>Marc Delmotte<br>Michel Ramonet<br>Olivier Laurent
PAL	Pallas-Sammaltunturi, GAW Station	67.9733	24.1157	Juha Hatakka<br>Tuula Aalto

Table S2: Atmospheric sites for which data was made available for the drought period via the ICOS atmospheric observations network.

Site code	Site name	Latitude (°N)	Longitude (°E)	Site Contacts
PDM	Pic du Midi	42.9372	0.1411	Marc Delmotte<br>Michel Ramonet<br>Francois Gheusi
PRS	Plateau Rosa Station	45.93	7.7	Francesco Apadula
PUI	Puijo	62.9096	27.6549	Juha Hatakka<br>Ari Leskinen
PUY	Puy de Dôme	45.7719	2.9658	Morgan Lopez<br>Camille Yver Kwok<br>Michel Ramonet<br>Aurélie Colomb<br>Marc Delmotte<br>Jean-Marc Pichon
RGL	Ridge Hill	51.9976	-2.54	Simon ODoherty<br>Dan Say
SAC	Saclay	48.7227	2.142	Michel Ramonet<br>Marc Delmotte
SMR	Hyytiälä	61.8474	24.2947	Petri Keronen<br>Ivan Mammarella
SSL	Schauinsland,<br>Baden-Wuerttemberg	47.92	7.92	Frank Meinhardt
SVB	Svartberget	64.256	19.775	Jutta Holst<br>Per Marklund<br>Mikaell Ottosson-Löfvenius
TAC	Tacolneston	52.5177	1.1386	Simon ODoherty<br>Dan
TRN	Trainou	47.9647	2.1125	Morgan Lopez<br>Camille Yver Kwok<br>Michel Ramonet<br>Marc Delmotte

Table S2: Atmospheric sites for which data was made available for the drought period via the ICOS atmospheric observations network.

Site code	Site name	Latitude (°N)	Longitude (°E)	Site Contacts
UTO	Utö - Baltic sea	59.7839	21.3672	Juha Hatakka<br>Tuomas Laurila
WAO	Weybourne, Norfolk	52.9502	1.1219	Andrew Manning<br>Grant Forster
ZEP	Ny-Alesund, Svalbard	78.9067	474.0	Cathrine Lund Myhre<br>Ove Hermansen

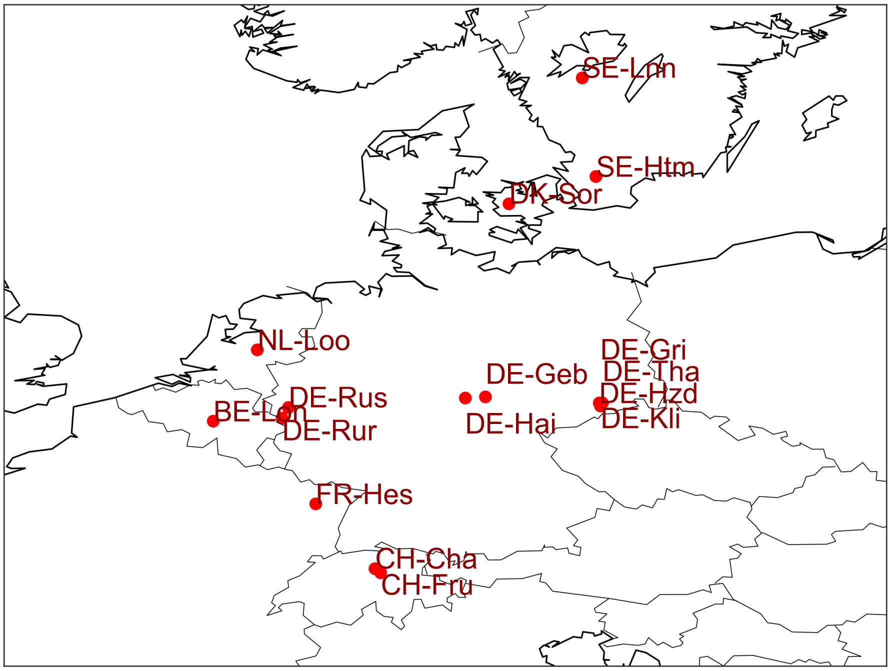

Figure S2: Overview of the locations of the used ICOS ecosystem sites within the drought region. See also Table S1 for more information on the sites.

C Correlation between remote sensing products and eddy fluxes

The spatial resolution of SIF means that it suffers from the mixed pixel problem, i.e. that there are usually multiple types of vegetation in each pixel so we cannot isolate the GPP response of a specific plant functional type or ecosystem measurement site. It also contains high noise-to-signal ratios at high latitudes. The higher spatial resolution of NIRv means that it goes a

long way in addressing this issue, but it is a measure of processes parallel to photosynthesis rather than of photosynthesis itself. It is therefore important to validate SIF and NIRv against measured rates of photosynthetic activity. In Table S3 we show the correlation coefficients, the gradient of the line of best linear fit, and the standard error of that gradient between the eddy covariance measurements of GPP from the selected ICOS ecosystem sites and SIF and NIRv in the corresponding pixels for 2013-2018. NIRv matches the observations very well, with correlation coefficients mostly in the range of 0.912-0.979, with only that at DE-Hai falling below 0.9 (though still high at 0.756.) SIF has slightly lower correlation coefficients in the range 0.624-0.958. This is most likely because of the coarser spatial resolution of SIF; it is likely better suited for looking at spatially integrated anomalies than site-level dynamics. We conclude that NIRv captures the behaviour of GPP at the ecosystem site level much better, and that we can reasonably assume that it can also be used as a proxy for GPP at also the regional scale beyond these ecosystem sites.

Table S3: Correlation coefficients, and gradients and standard deviations thereof of the lines of best fit between SIF and NIRv-derived GPP and GPP from eddy covariance measurements at the 15 selected sites for the period 2013-2018. SIF and NIRv are calculated for the pixel containing each measurement site (0.5 degrees for SIF and 0.05 degrees for NIRv), so cover a broader footprint than the site alone.

site name	NIRv corr	NIRv slope	NIRv slope stderr	SIF corr	SIF slope	SIF slope stderr
BE-Lon	0.950	0.017	0.000	0.784	0.072	0.005
DE-Hzd	0.956	0.014	0.000	0.624	0.087	0.014
DE-Kli	0.912	0.016	0.001	0.792	0.091	0.007
DE-Tha	0.971	0.024	0.000	0.713	0.141	0.011
DE-Gri	0.967	0.015	0.000	0.690	0.112	0.011
DE-Hai	0.756	0.013	0.001	0.667	0.082	0.008
SE-Htm	0.948	0.016	0.001	0.820	0.123	0.013
SE-Lnn	0.979	0.023	0.001	0.860	0.112	0.010
DE-Rus	0.961	0.012	0.000	0.783	0.092	0.010
DE-Geb	0.971	0.020	0.000	0.958	0.153	0.004
FR-Hes	0.932	0.013	0.001	0.885	0.077	0.006
NL-Loo	0.950	0.010	0.000	0.670	0.095	0.008
DK-Sor	0.943	0.012	0.000	0.787	0.061	0.004
DE-Rur	0.932	0.021	0.001	0.761	0.117	0.013
CH-Cha	0.928	0.014	0.000	0.957	0.129	0.004
CH-Fru	0.966	0.017	0.000	0.885	0.081	0.004

We use these correlations to upscale NIRv and SIF to make regional GPP estimates. Figure S3 shows GPP and its anomalies derived from upscaling NIRv and SIF in this way for grasslands, croplands, forests, and all other plant functional types.

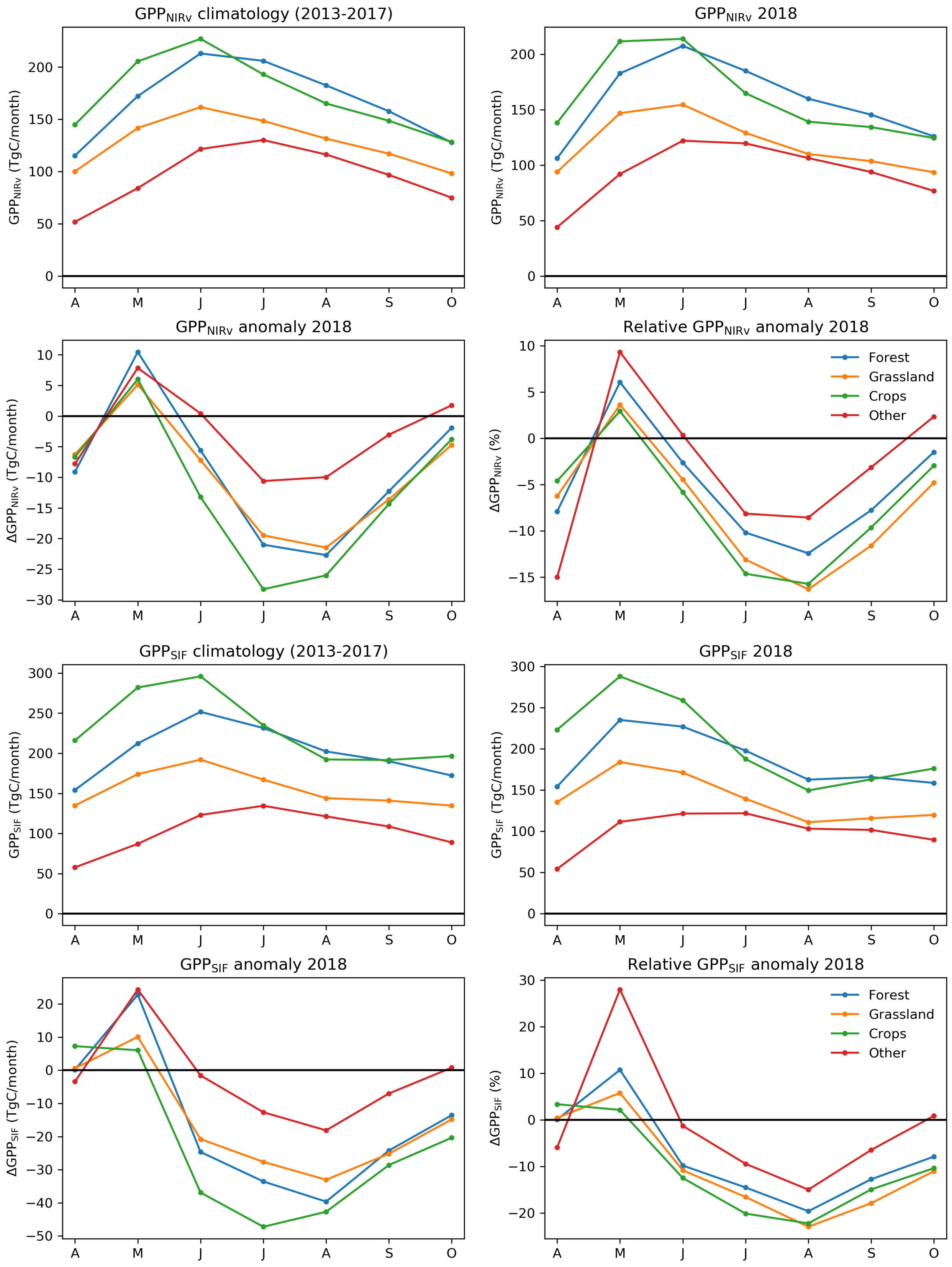

Figure S3: GPP from upscaled NIRv (top four) and SIF (bottom four) for each of the four considered plant functional types. Of the four subplots, the upper left shows the GPP climatology for 2013-2017, the upper right shows the GPP during 2018, the lower left shows the anomaly in GPP during 2018 compared to the climatology, and the lower right shows the corresponding relative anomaly.

D SiB4 runs

Figure S4 shows 2018 anomalies of GPP, TER, NEE, and LE of the ENF biome for four SiB4 runs, using: the default SiB4 settings, SiB4 with modified rooting depth (the run used in the main text analysis), SiB4 with the upper layers' soil moisture given by PCR-GLOBWB, and SiB4 with the modified rooting depth for ENF biomes and a removal of the 0.7 lower bound on the ENF biome stress factors.

All three modifications appear to improve SiB4's representation of the drought anomaly in the four parameters, with the change in rooting depth and removal of the 0.7 threshold making the largest differences to the magnitude of the anomaly.

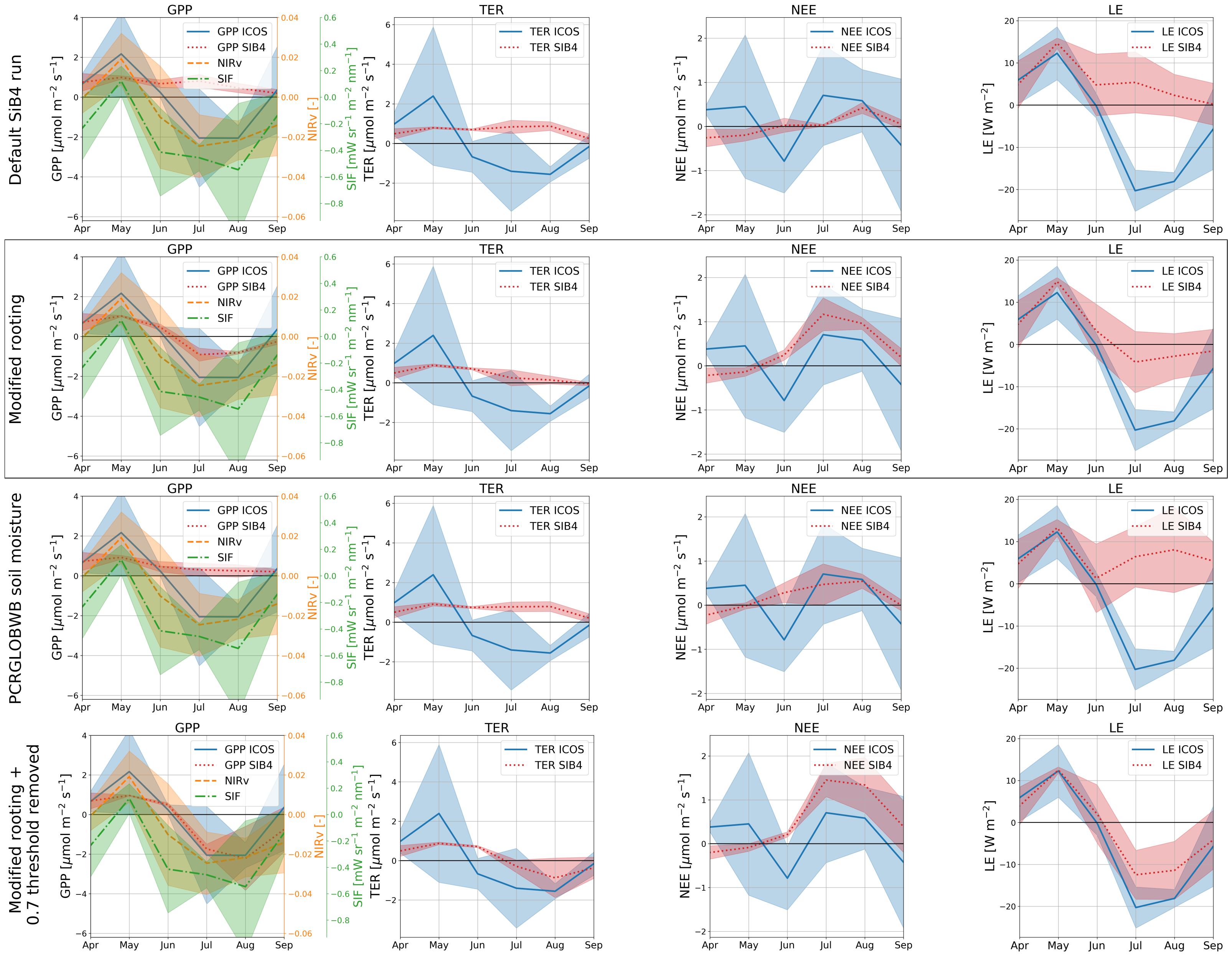

Figure S4: SIF, NIRv and modelled and measured NEE, GPP, TER, and LE anomalies of 2018 against the climatological average (2013-2017) of the 3 evergreen needleleaf sites for four different SiB4 runs from top to bottom: the default SiB4 run; the SiB4 run with modified rooting depth as used in the main text and therefore highlighted with a frame; the SiB4 run with soil moisture replaced by PCRGLOB-WB and the SiB4 run with modified rooting depth and the soil moisture stress factor threshold of 0.7 removed. The average over the different sites is shown together with the 1-σ spread around the mean. The modelled GPP (SiB4) represents the same PFT as that at the measurement location. SIF and NIRv products are taken from the satellite pixel in which the ICOS measurement site is located.

E Soil moisture in SiB4 and using PCR-GLOBWB

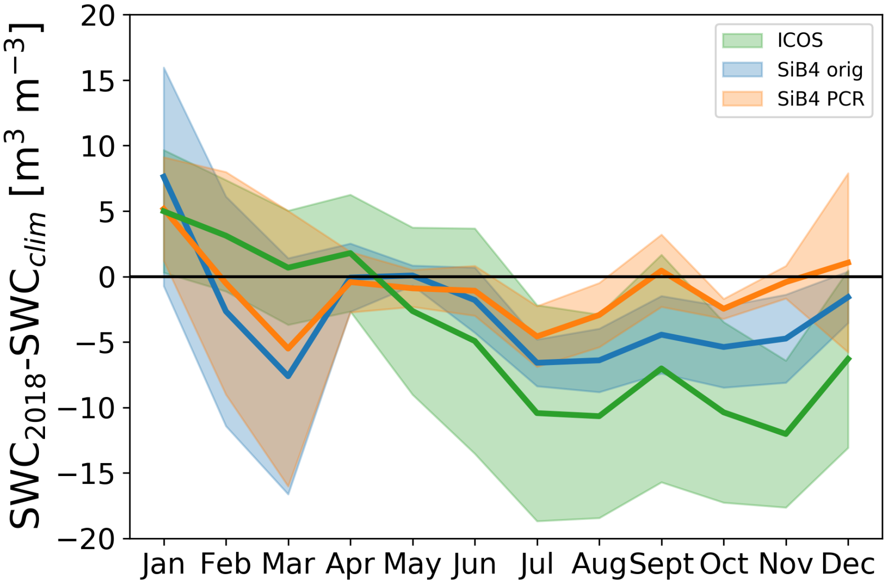

Figure S5: Comparison of the observed and simulated soil water content (SWC) at ICOS Ecosystem sites. SWC was simulated with the default SiB4 version as well as SiB4 with soil moisture replaced by PCR-GLOBWB. Simulated soil moisture is represented by the average over three SiB4 soil layers closest to the surface (0-0.28 m), which links to the depth of the upper PCR-GLOBWB soil moisture layer of 0-0.3 m. Observed ICOS SWC represents measurements at 0.05 m depth.

Soil moisture is known to be a very difficult parameter to model accurately. To better understand the uncertainty of the stress that the plants experience in the biosphere model SiB4, we carried out a second simulation replacing the internally calculated soil moisture with that of the more advanced hydrological model PCR-GLOBWB. Figure S5 shows the soil water content (SWC) for these two simulations, as well as that from observations at ICOS ecosystem sites. It should be noted that the vertical resolutions of these three products are not identical; we look the mean for the three surface layers of SiB4 (corresponding to depths of 0-0.28 m), the uppermost of the two layers used in PCR-GLOBWB (0-0.3 m), and measured soil moisture at a depth of 0.05 m. PCR-GLOBWB was also driven using ERA5 data, whereas SiB4 was driven using MERRA-2. The temporal variability of SWC is consistent between these three products, and the magnitudes are similar throughout the year, with both modelled products falling mostly within the range of the observations. The run using PCR-GLOBWB typically yielded slightly higher soil water content than that produced by the hydrological component of SiB4, and indeed the soil moisture stress factor was slightly higher in this setup (and thus vegetation experienced slightly lower soil moisture stress) during August as shown in Figure S6.

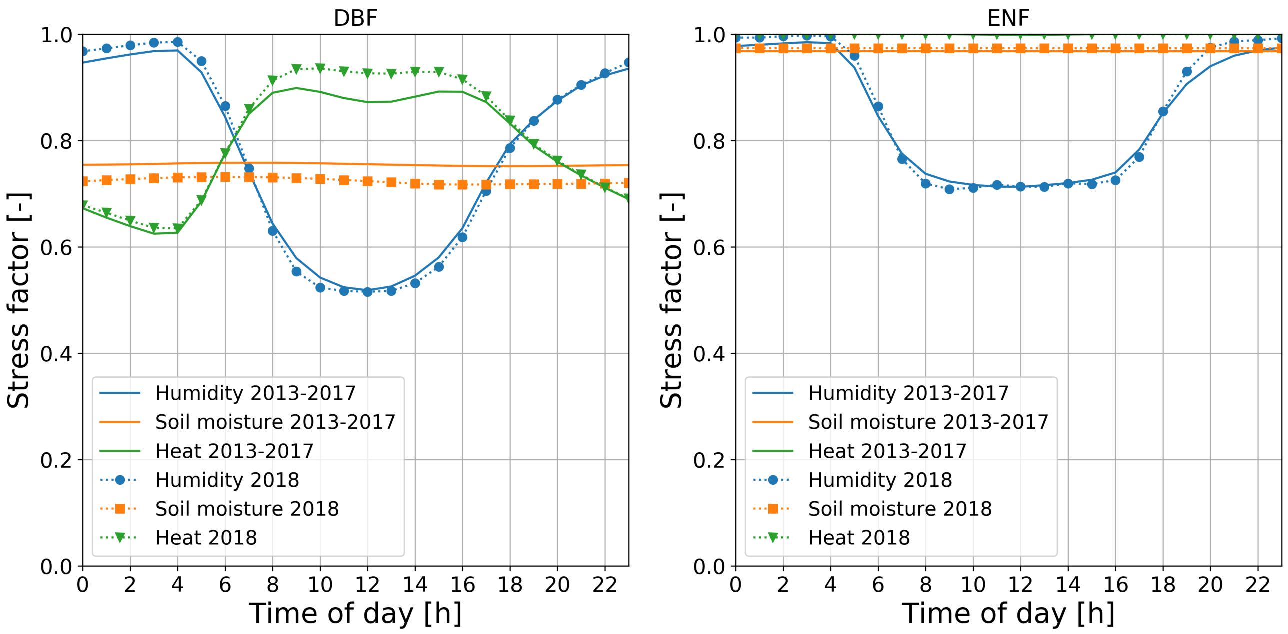

Figure S6: Diurnal cycle of simulated stress factors experienced by (left) deciduous broadleaf forest and (right) evergreen needleleaf forest plant functional types for meteorological conditions representative for the German ecosystem site Hainich during the month August in the simulation using soil moisture fields from PCR-GLOBWB. The lowest line indicates that this stress factor was the one limiting photosynthesis at that point in time, with green corresponding to heat stress, blue to humidity stress, and orange to soil moisture stress. Solid lines show the climatological mean for 2013-2017 and dotted lines to 2018.

F Biomass burning

The drought-affected region typically experiences small emissions from wildfire burning of biomass. Throughout the summer of 2018, uncharacteristically large wildfire events occurred across the south of Sweden, reflected in the numerous strong peaks in the biomass burning emissions of Figure S7. Compared to 2013-2017 (excluding one large event in 2014), these peaks regularly rise well above those typical of the preceding five years.

G Extended anomalies of 2018 against the climatological average

In the main text, we show the progression of the monthly mean anomalies in NEE, GPP, TER, and SWC from SiB4 and eddy covariance measurements, alongside those of SIF- and NIR-derived GPP, for 2018 compared to 2013-2017 at forest ecosystem sites. Here, in Figure S2 we show these same quantities for the (1) grassland and (2) crop sites. The anomalies in the measured quantities at grassland sites are well captured by SiB4, matching the timing and

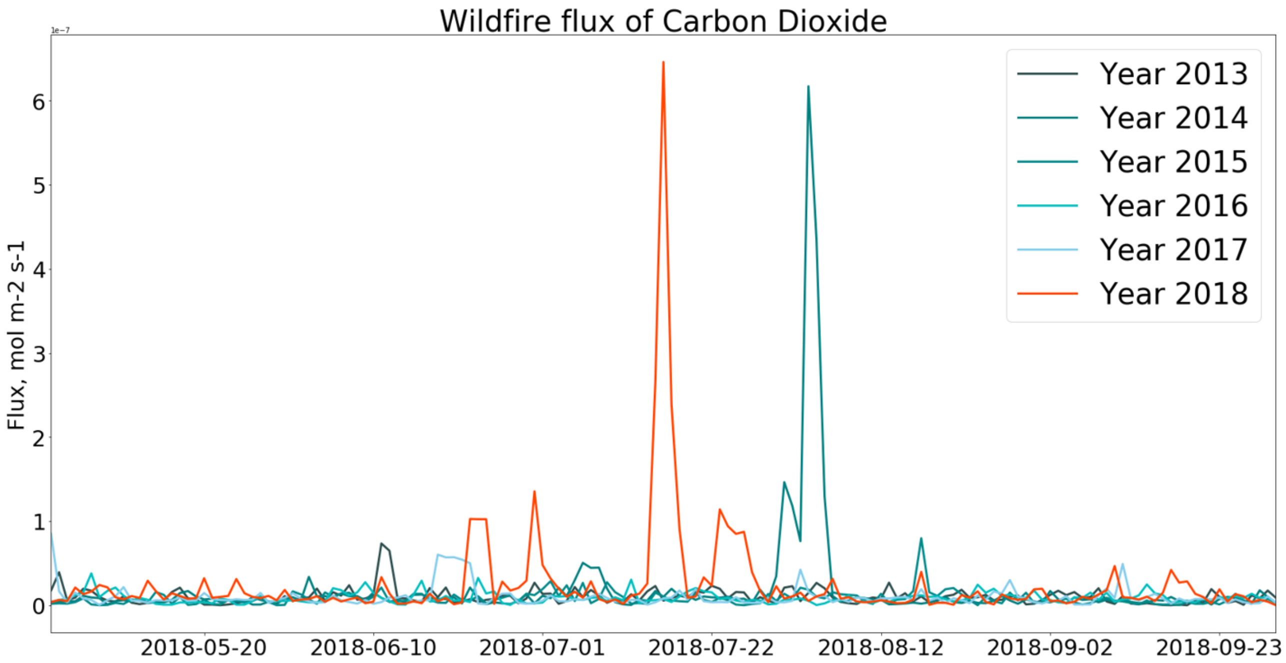

Figure S7: Daily wildfire biomass burning emissions from GFAS over the most severely effected region. 2018 is shown in orange, with large peaks corresponding to wildfire events in the south of Sweden. The area saw fewer (and mostly smaller magnitude) fire events in the previous five years.

magnitude of the measured anomalies well in all parameters except perhaps soil water content. At the cropland sites we see a poorer match to these characteristics, possibly due to the rotation of different crops throughout the years in reality rather than the consistent use of one type of plant, as was the case in SiB4, or due to agricultural practices that are not accounted for in SiB4. Note that we are also missing soil water content measurements for some months at the cropland sites.

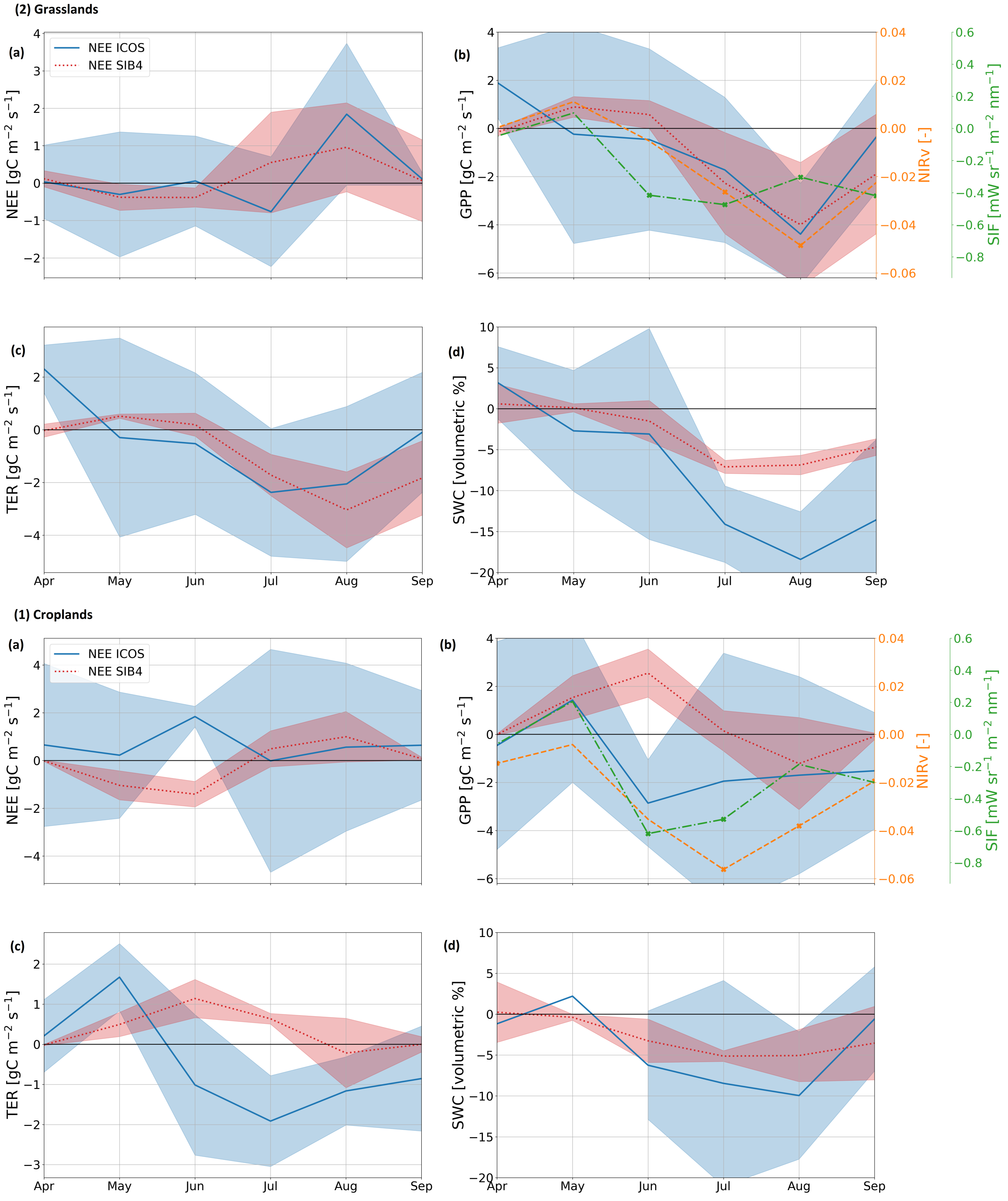

Figure S8: SIF, NIRv and modelled and measured NEE (a), GPP (b), TER (c), and SWC (d) anomalies of 2018 against the climatological average (2013-2017) for grasslands (1) and crops (2). The average over the different sites is shown together with the 1-σ spread around the mean. The modelled GPP (SiB4) represents the same PFT as that at the measurement location. SIF and NIRv products are taken from the satellite pixel in which the ICOS measurement site is located and anomalies that exceed 1-σ are indicated with a square symbol. Soil moisture is derived from ecosystem site measurements taken in the top 0.05 m of the soil and from the uppermost three layer(s) of the hydrological component of SiB4.

Table S4: Anomalies of the measured and simulated carbon fluxes in 2018 compared to the climatology of 2013-2017 for the months July, August and September. Values calculated based on monthly data and standard deviations of the climatology are given in brackets.

sitename	PFT	GPP ICOS (σ)	TER ICOS (σ)	NEE ICOS (σ)	GPP SIB4 (σ)	TER SIB4 (σ)	NEE SIB4 (σ)
DE-Hai	Deciduous Broadleaf Forest	-6.61 (2.06)	-2.01 (0.14)	4.44 (1.61)	-3.49 (0.25)	-2.82 (0.67)	0.68 (0.42)
DE-Hzd	Deciduous Broadleaf Forest	-1.29 (0.96)	-4.42 (0.84)	-3.12 (0.58)	-3.80 (1.93)	-2.66 (1.71)	1.14 (0.45)
DK-Sor	Deciduous Broadleaf Forest	-2.87 (3.71)	-1.89 (2.35)	1.02 (1.36)	-0.94 (0.75)	-0.81 (0.47)	0.13 (0.34)
FR-Hes	Deciduous Broadleaf Forest	-0.45 (1.65)	1.03 (0.99)	1.47 (0.90)	-2.07 (1.54)	-1.70 (1.07)	0.38 (0.58)
DE-Tha	Evergreen Needleleaf Forest	-2.33 (0.92)	-1.05 (0.72)	1.35 (0.53)	-0.65 (0.18)	0.16 (0.18)	0.81 (0.34)
NL-Loo	Evergreen Needleleaf Forest	-1.69 (0.58)	-1.02 (0.07)	0.66 (0.34)	-1.18 (0.35)	-0.11 (0.04)	1.07 (0.31)
SE-Htm	Evergreen Needleleaf Forest	-1.75 (3.42)	-1.77 (1.92)	0.14 (1.47)	-0.48 (0.39)	0.35 (0.44)	0.83 (0.82)
CH-Cha	C3 Grassland	-3.38 (1.72)	-1.90 (0.07)	1.29 (1.56)	-2.88 (1.80)	-2.40 (1.31)	0.48 (0.67)
CH-Fru	C3 Grassland	-0.43 (3.37)	0.97 (2.51)	0.56 (3.06)	-2.66 (2.00)	-2.27 (1.46)	0.39 (0.69)
DE-Gri	C3 Grassland	-4.56 (2.68)	-4.28 (2.54)	0.31 (0.61)	-4.82 (1.16)	-3.08 (0.82)	1.74 (0.57)
DE-RuR	C3 Grassland	-0.25 (1.61)	-0.83 (1.09)	-0.58 (0.92)	-4.94 (1.25)	-3.28 (0.79)	1.65 (0.59)
BE-Lon	C3 Cropland	-2.51 (0.76)	-1.48 (0.56)	1.00 (1.20)	0.38 (0.37)	0.47 (0.30)	0.09 (0.10)
DE-Geb	C3 Cropland	-1.41 (1.22)	-0.90 (0.23)	0.52 (1.01)	-1.89 (2.39)	-0.70 (0.79)	1.20 (1.60)
DE-Kli	C3 Cropland	3.06 (3.56)	-1.41 (1.12)	-4.48 (2.56)	-1.36 (1.44)	-0.24 (0.76)	1.11 (0.98)
DE-RuS	C3 Cropland	-6.03 (0.70)	-2.24 (1.35)	3.76 (0.83)	0.31 (0.28)	0.46 (0.27)	0.14 (0.05)
SE-Lnn	C3 Cropland	-1.53 (3.26)	-0.19 (1.13)	1.36 (2.20)	-1.36 (1.72)	-0.27 (0.90)	1.09 (1.09)

References

[1] Bastos A, Ciais P, Friedlingstein P, Sitch S, Pongratz J, Fan L, et al. Direct and seasonal legacy effects of the 2018 heat and drought on European ecosystem productivity. Science Advances. 2020;.

[2] Quiring SM. Developing objective operational definitions for monitoring drought. Journal of Applied Meteorology and Climatology. 2009;48(6):1217–1229.

[3] Knohl A, Schulze ED, Kolle O, Buchmann N. Large carbon uptake by an unmanaged 250-year-old deciduous forest in Central Germany. Agricultural and Forest Meteorology. 2003;118(3-4):151–167.

[4] Prescher AK, Grünwald T, Bernhofer C. Land use regulates carbon budgets in eastern Germany: From NEE to NBP. Agricultural and Forest Meteorology. 2010;150(7-8):1016–1025.

[5] Pilegaard K, Ibrom A, Courtney MS, Hummelshøj P, Jensen NO. Increasing net CO2 uptake by a Danish beech forest during the period from 1996 to 2009. Agricultural and Forest Meteorology. 2011;151(7):934–946.

[6] Wu J, Larsen KS, van der Linden L, Beier C, Pilegaard K, Ibrom A. Synthesis on the carbon budget and cycling in a Danish, temperate deciduous forest. Agricultural and Forest Meteorology. 2013;181:94–107.

[7] Granier A, Bréda N, Longdoz B, Gross P, Ngao J. Ten years of fluxes and stand growth in a young beech forest at Hesse, North-eastern France. Annals of Forest Science. 2008;65(7):1.

[8] Borchard N, Schirrmann M, von Hebel C, Schmidt M, Baatz R, Firbank L, et al. Spatio-temporal drivers of soil and ecosystem carbon fluxes at field scale in an upland grassland in Germany. Agriculture, ecosystems & environment. 2015;211:84–93.

[9] Zeeman MJ, Hiller R, Gilgen AK, Michna P, Plüss P, Buchmann N, et al. 10 Appendix A: Management, not climate, controls net CO2 fluxes and carbon budgets of three grasslands along an elevational gradient in Switzerland. Drought in Swiss grasslands at different altitudes: Effects on productivity and resource use;p. 161.

[10] Hörtnagl L, Barthel M, Buchmann N, Eugster W, Butterbach-Bahl K, Díaz-Pinés E, et al. Greenhouse gas fluxes over managed grasslands in Central Europe. Global Change Biology. 2018;24(5):1843–1872.

[11] Elbers JA, Jacobs CM, Kruijt B, Jans WW, Moors EJ. Assessing the uncertainty of estimated annual totals of net ecosystem productivity: A practical approach applied to a mid latitude temperate pine forest. Agricultural and forest meteorology. 2011;151(12):1823–1830.

[12] Delin E. Forest Reaction to the 2018 Drought: Comparing the Hyltemossa and Rumperöd Forests. Student thesis series INES. 2019;.

[13] Buysse P, Manise T, De Ligne A, Moureaux C, Bodson B, Heinesch B, et al. Climatic and management drivers of CO2 exchanges by a production crop: Analysis over three successive 4-year crop rotation cycles. 2016;.

[14] Anthoni PM, Freibauer A, Kolle O, Schulze ED. Winter wheat carbon exchange in Thuringia, Germany. Agric For Meteorol. 2004;121.1-2:55–67.

[15] Hurkuck M, Brümmer C, Kolle O, Kutsch WL, Lucas-Moffat AM, Mukwashi K, et al. Long-term net carbon measurements over three full crop rotations. In preparation;.
